# Supplementary material for: Testing strategies to detect acute and prevalent HIV infection in adult outpatients seeking healthcare for symptoms compatible with acute HIV infection in Kenya: a cost-effectiveness analysis
Source: BMJ Open. 2022 Sep 28;12(9):e058636. doi: 10.1136/bmjopen-2021-058636 (PMC9528633; doi:10.1136/bmjopen-2021-058636)
Supplement: Supplementary data [file bmjopen-2021-058636supp001.pdf]

BMJ Publishing Group Limited (BMJ) disclaims all liability and responsibility arising from any reliance placed on this supplemental material which has been supplied by the author(s)

Time and Motion Survey

Date: |__ __|__ __|__ __ __ __|

Clinic: |____

Patient #: |__ __ __|

Note: Please use the 24 hour clock system (use time piece with 24-hour clock for this study)

#	Point of patient contact	Time in	Time out	Signature
1	**Patient enters clinic**<br>Received by (initial): |__________|	|___ ___:___ ___|		
2	**Patient seen by (designation):** |_________________| Initial: |__________|	|___ ___:___ ___|	|___ ___:___ ___|	
3	**Patient seen by (designation):** |_________________| Initial: |__________|	|___ ___:___ ___|	|___ ___:___ ___|	
4	**Patient seen by (designation):** |_________________| Initial: |__________|	|___ ___:___ ___|	|___ ___:___ ___|	
5	**Patient seen by (designation):** |_________________| Initial: |__________|	|___ ___:___ ___|	|___ ___:___ ___|	
6	**Patient seen by (designation):** |_________________| Initial: |__________|	|___ ___:___ ___|	|___ ___:___ ___|	
7	**Patient seen by (designation):** |_________________| Initial: |__________|	|___ ___:___ ___|	|___ ___:___ ___|	
8	**Patient seen by (designation):** |_________________| Initial: |__________|	|___ ___:___ ___|	|___ ___:___ ___|	
9	**Patient seen by (designation):** |_________________| Initial: |__________|	|___ ___:___ ___|	|___ ___:___ ___|	
15	**Patient leaves clinic**<br>Seen off by (initial): |__________|		|___ ___:___ ___|	
