# Supplementary material for: Testing strategies to detect acute and prevalent HIV infection in adult outpatients seeking healthcare for symptoms compatible with acute HIV infection in Kenya: a cost-effectiveness analysis
Source: BMJ Open. 2022 Sep 28;12(9):e058636. doi: 10.1136/bmjopen-2021-058636 (PMC9528633; doi:10.1136/bmjopen-2021-058636)
Supplement: Author's manuscript [file bmjopen-2021-058636.draft_revisions.pdf]

BMJ Open is committed to open peer review. As part of this commitment we make the peer review history of every article we publish publicly available.

When an article is published we post the peer reviewers' comments and the authors' responses online. We also post the versions of the paper that were used during peer review. These are the versions that the peer review comments apply to.

The versions of the paper that follow are the versions that were submitted during the peer review process. They are not the versions of record or the final published versions. They should not be cited or distributed as the published version of this manuscript.

BMJ Open is an open access journal and the full, final, typeset and author-corrected version of record of the manuscript is available on our site with no access controls, subscription charges or pay-per-view fees (http://bmjopen.bmj.com).

If you have any questions on BMJ Open's open peer review process please email info.bmjopen@bmj.com

BMJ Open

Testing strategies to detect acute and prevalent HIV infection in adult outpatients seeking healthcare for symptoms compatible with acute HIV infection in Kenya: a cost-effectiveness analysis

Journal:	*BMJ Open*
Manuscript ID	bmjopen-2021-058636
Article Type:	Original research
Date Submitted by the Author:	24-Oct-2021

Complete List of Authors:	Babigumira, Joseph; National University Singapore Saw Swee Hock School of Public Health; National University Health System,<br>Agutu, Clara A.; KEMRI-Wellcome Trust Research Programme<br>Hamilton, Deven T.; University of Washington<br>van der Elst, Elise; KEMRI-Wellcome Trust Research Programme<br>Hassan, Amin; KEMRI-Wellcome Trust Research Programme<br>Gichuru, Evanson; KEMRI-Wellcome Trust Research Programme<br>Mugo, Peter; KEMRI-Wellcome Trust Research Programme, Public health<br>Farquhar, Carey; University of Washington Department of Medicine<br>Ndung'u, Thumbi ; Africa Health Research Institute<br>Sirengo, Martin; National AIDS & STI Control Programme<br>Chege, Wairimu; National Institutes of Health<br>Goodreau, Steven M; University of Washington, Departments of Anthropology and Epidemiology<br>Sanders, Eduard; KEMRI - Wellcome Trust Research Programme; University of Oxford<br>Graham, Susan; KEMRI-Wellcome Trust Research Programme; University of Washington School of Medicine

Keywords:	Health economics < HEALTH SERVICES ADMINISTRATION & MANAGEMENT, Health policy < HEALTH SERVICES ADMINISTRATION & MANAGEMENT, HEALTH ECONOMICS, HIV & AIDS < INFECTIOUS DISEASES

SCHOLARONE™
Manuscripts

I, the Submitting Author has the right to grant and does grant on behalf of all authors of the Work (as defined in the below author licence), an exclusive licence and/or a non-exclusive licence for contributions from authors who are: i) UK Crown employees; ii) where BMJ has agreed a CC-BY licence shall apply, and/or iii) in accordance with the terms applicable for US Federal Government officers or employees acting as part of their official duties; on a worldwide, perpetual, irrevocable, royalty-free basis to BMJ Publishing Group Ltd ("BMJ") its licensees and where the relevant Journal is co-owned by BMJ to the co-owners of the Journal, to publish the Work in this journal and any other BMJ products and to exploit all rights, as set out in our licence.

The Submitting Author accepts and understands that any supply made under these terms is made by BMJ to the Submitting Author unless you are acting as an employee on behalf of your employer or a postgraduate student of an affiliated institution which is paying any applicable article publishing charge ("APC") for Open Access articles. Where the Submitting Author wishes to make the Work available on an Open Access basis (and intends to pay the relevant APC), the terms of reuse of such Open Access shall be governed by a Creative Commons licence – details of these licences and which Creative Commons licence will apply to this Work are set out in our licence referred to above.

Other than as permitted in any relevant BMJ Author's Self Archiving Policies, I confirm this Work has not been accepted for publication elsewhere, is not being considered for publication elsewhere and does not duplicate material already published. I confirm all authors consent to publication of this Work and authorise the granting of this licence.

For peer review only

Testing strategies to detect acute and prevalent HIV infection in adult outpatients seeking healthcare for symptoms compatible with acute HIV infection in Kenya: a cost-effectiveness analysis

Authors

Joseph B. Babigumira[1], Clara A. Agutu[2], Deven T. Hamilton[3], Elise van der Elst[2], Amin Hassan[2], Evanson Gichuru[2]; Peter Mugo[2], Carey Farquhar[4], Thumbi Ndung'u[5], Martin Sirengo[6], Wairimu Chege[7], Steven M. Goodreau[8], Eduard J. Sanders[2,9], Susan M. Graham[2,4]

1. Saw Swee Hock School of Public Health, National University of Singapore and National University Health System, Singapore
2. KEMRI - Wellcome Trust Research Programme, Kilifi, Kenya
3. Center for Studies in Demography & Ecology, University of Washington, Seattle, WA, United States
4. Departments of Medicine, Global Health, and Epidemiology, University of Washington, Seattle, WA, United States
5. Africa Health Research Institute, Durban, South Africa
6. National AIDS & STI Control Programme, Nairobi, Kenya
7. National Institute of Allergy & Infectious Diseases, National Institutes of Health, Rockville, MD, United States
8. Departments of Anthropology and Epidemiology, University of Washington, Seattle, WA, United States
9. University of Oxford, Headington, United Kingdom

Corresponding author

Joseph B. Babigumira
Saw Swee Hock School of Public Health
National University of Singapore
babijo@nus.edu.sg

For peer review only - http://bmjopen.bmj.com/site/about/guidelines.xhtml

Abstract

Background: Detection of acute and prevalent HIV infection using point-of-care nucleic acid amplification testing (POC-NAAT) among outpatients with symptoms compatible with acute HIV is critical to HIV prevention but it is not clear if it is cost-effective compared to existing HIV testing strategies.

Methods: We developed and parametrized a decision tree to compare the cost-effectiveness of: (1) provider-initiated testing and counseling (PITC) using rapid tests, the standard of care; (2) expanded PITC (E-PITC) in which all patients were tested with rapid tests unless they opted out; and (3) opt-out testing and counseling using POC-NAAT, which detects both acute and prevalent infection. We obtained data from the Tambua Mapema Plus randomized controlled trial of a POC-NAAT intervention in Kenya, supplemented with results from a stochastic, agent-based network model of HIV-1 transmission to evaluate population-level and HIV prevention impact. The analysis was conducted from the perspective of the Kenyan government using a primary outcome of cost per DALY averted over a 10-year time horizon.

Results: The average per patient cost of POC-NAAT was \$214.9 compared with \$173.6 for E-PITC and \$47.3 for PITC. The mean DALYs accumulated per patient for POC-NAAT were 0.160 compared with 0.176 for E-PITC and 0.214 for PITC. In the incremental analysis, E-PITC was eliminated due to extended dominance and the ICER comparing POC-NAAT to PITC was \$3,098 per DALY averted. The ICER was sensitive to disability weights for HIV/AIDs and the costs of antiretroviral therapy.

Conclusion: Expanded POC-NAAT offered to adult outpatients in Kenya who present for care with symptoms compatible with AHI is cost-effective and should be considered for inclusion as the standard of HIV testing in this population.

For peer review only - http://bmjopen.bmj.com/site/about/guidelines.xhtml

Strengths and limitations of this study

- This cost-effectiveness analysis was comprehensive because it modeled the short-term clinical and long-term prevention impact of testing for both acute and chronic HIV using nucleic acid amplification testing (NAAT) in symptomatic patients at out-patient facilities Kenya.
- We combined data from a randomized controlled study (short-term clinical impact) and a stochastic network-based mathematical model (long-term prevention impact) to estimate both length of life and disability.
- This is the first study in sub-Saharan Africa to consider the cost-effectiveness of NAAT testing for HIV including acute HIV infection among symptomatic outpatients.
- The study was limited by the use of the relatively short time horizon of 10 years and not a lifetime horizon owing to uncertainty in the impact of HIV testing on outcomes long into the future.
- The study is generalizable to and therefore useful for policy making in other, similar countries in sub-Saharan Africa with high HIV prevalence and populations vulnerable to HIV as assessed by risk scoring at outpatient health facilities.

For peer review only

For peer review only - http://bmjopen.bmj.com/site/about/guidelines.xhtml

Introduction

HIV testing is the gateway to lifelong treatment of HIV-positive individuals with antiretroviral therapy (ART), which prolongs life, reduces morbidity, and optimizes quality of life. HIV testing is also the gateway to HIV prevention through knowledge of HIV status, which empowers HIV-negative individuals to protect themselves, and to treatment as prevention, which effectively renders treated individuals who achieve virologic suppression unable to transmit to others.[1] In 2015, UNAIDS set a target of achieving a 90% rate of diagnosis of people living with HIV (PLWH) by 2020 and a 95% rate of diagnosis of PLWH by 2030, and put both metrics on a “fast track” to ending the AIDS epidemic as a public health threat.[2] However, global progress has not achieved the desired goals: only 81% percent of PLWH knew their status in 2019.[3]

Although Kenya achieved the 90% diagnosis goal for PLWH by the end of 2020[3], reaching the 95% target will be challenging because the current HIV rapid antibody detection tests in use in Kenya are unable to diagnose acute HIV infection (AHI), present during the period before seroconversion[4] In Kenya, most patients with AHI develop a febrile illness and present to health facilities for urgent care.[5] This provides an opportunity for early diagnosis of AHI at outpatient clinics using newly available nucleic acid amplification testing (NAAT) technologies.[6] Diagnosis of AHI with linkage to care and prompt treatment with ART would lead to important downstream treatment and prevention benefits. Testing for AHI has significant HIV prevention implications because the AHI period is a period of high risk for secondary HIV transmission due to very high viral load.[7] While there are advantages of NAAT in outpatient health facilities in resource-limited settings, these tests remain costly, sometimes up to 20 times the cost of rapid tests on a per-test basis. Consequently, there has been a lack of effectiveness and cost-effectiveness evidence to support the widespread use of NAAT testing in the outpatient setting in Kenya and other sub-Saharan African countries.

To address the effectiveness gap in knowledge, the Tambua Mapema Plus (TMP) study, a modified stepped wedge randomized controlled trial, was conducted. The TMP trial assessed the yield of an opt-out point-of-care (POC) NAAT intervention (POC-NAAT) using Cepheid’s Xpert® HIV-1 Qual to diagnose acute and chronic HIV infections in Kenya.[8] In the trial, use of opt-out POC-NAAT resulted in a two-fold greater odds of HIV diagnosis compared to standard of care, i.e. provider-initiated HIV-1 antibody testing.[9] The TMP trial also provided the parameterization basis for the development of a stochastic, agent-based network model to simulate the HIV epidemic in Kenya and quantify the population-level prevention benefits of POC-NAAT testing.[8] The modeling study included an expanded provider-initiated testing strategy in which all eligible outpatients were modeled to receive rapid HIV tests unless they opted out. The model projected that using opt-out POC-NAAT was superior to provider-initiated testing or expanded HIV rapid testing in terms of both yield of HIV-positives (knowledge of status) and HIV prevention.[10] In this study, we present the results of a cost-effectiveness analysis to address the gap in economic evidence to further support the widespread use of NAAT testing for AHI in the outpatient setting in Kenya and other sub-Saharan African countries.

Methods

For peer review only - http://bmjopen.bmj.com/site/about/guidelines.xhtml

Study design. We conducted a model-based cost-effectiveness analysis using costs and outcomes data obtained from the TMP trial.[8 9] Additional outcome data were obtained from the stochastic, network-based model of HIV-1 transmission that was parameterized using data from the TMP trial.[8] We also used data from the published literature to supplement cost and outcome data obtained from the trial and outcome data obtained from the stochastic, network-based model.

Target population. The base case population in the analysis included adults 18–39 years, not previously diagnosed with HIV, who presented as outpatients to six public and private primary care health facilities with one or more symptoms of AHI. Patients qualified for inclusion in the trial, and consequently in this study, if they had a score of ≥2 on a risk screening score defined by younger age (18–29 years), fever, fatigue, body pains, diarrhea, and sore throat (1 point each), or genital ulcer disease (3 points).[8 11]

Setting. The study setting and decision-making context was peri-urban coastal Kenya (Mombasa and Kilifi counties), an area with busy nightlife, sex work, and tourism (estimated HIV prevalence of 5.6% and 2.3% for Mombasa and Kilifi counties respectively in 2018.[12]) Therefore, insights from this study may also have decision-making relevance in urban and peri-urban settings in Kenya and other, similar low-income, high HIV burden countries.

Comparators. We compared three HIV testing strategies as follows:

1. Provider-initiated HIV-1 testing and counseling (PITC), the standard of current care in Kenya. Under PITC, providers ordered rapid HIV-1 antibody testing at their discretion as a diagnostic aid. This comparator is identical to the observation arm in the TMP trial, in which only 352 (25.6%) of 1374 enrolled participants were tested for HIV and 13 (0.9%) of 1374 enrolled participants were newly diagnosed with HIV.[13]
2. Enhanced PITC (E-PITC), in which providers were modeled to order rapid HIV-1 antibody testing for all patients unless they opted out of HIV testing. This comparator was included as a potentially plausible policy alternative for testing among this group of outpatients, given that it excludes relatively costly POC-NAAT.
3. POC-NAAT testing and counseling using point-of-care Xpert® HIV-1 Qual, as performed under the intervention arm of the TMP trial. Under this POC-NAAT approach, providers ordered NAAT testing for all included patients except those who were eligible but opted out of the study. Patients who tested positive by POC-NAAT received rapid HIV-1 antibody testing to distinguish between acute and prevalent HIV infection. This comparator is identical to the intervention arm in the TMP trial, in which all 1,500 participants were tested for HIV, and 37 (2.5%) were newly diagnosed with HIV, of whom 2 were AHI cases.[9]

Time horizon. The analysis was conducted over a time horizon of 10 years, consistent with the mathematical model[10], and thereby included the 10-year costs of ART for individuals that tested positive for HIV and costs of pre-exposure prophylaxis (PrEP) for HIV-negative partners of newly diagnosed index patients that were based on the mean duration of PrEP use in Kenya (Table 1).

For peer review only - http://bmjopen.bmj.com/site/about/guidelines.xhtml

Decision analytic model. We developed a decision-analytic model (decision tree) to assess the cost-effectiveness of the three different HIV testing strategies (Figure 1). The data used to parameterize the model, including ranges used for sensitivity analysis, are shown in detail in Table 1. In PITC, base-case patients received a Determine® HIV antibody test at the recommendation and discretion of the provider unless they refused HIV testing. In E-PITC, all patients received a Determine® HIV antibody test unless they opted out. Patients who tested positive with Determine® in either scenario received confirmatory testing with the First Response® HIV antibody test. In POC-NAAT, patients who had positive HIV-1 RNA Qual results received HIV rapid testing with the two different antibody tests in parallel, to distinguish acute from prevalent HIV infection. In each study arm, HIV rapid tests were repeated for confirmation of diagnosis prior to ART initiation, in accordance with Kenyan guidelines.[14]

The decision tree then divided patients into HIV-positive and HIV-negative based on the prevalence of HIV in Kenya.[12] The decision tree assessed the accuracy of testing strategies by dividing patient test results into true positive (TP), false negative (FN), false positive (FP) and true negative (TN), based on estimates from the mathematical model.[10] The decision tree simulated the experience of base-case patients and their likelihood of ending up in the four diagnostic accuracy groups, given modeled HIV transmission risk, rates of HIV rapid testing in the outpatient setting, and background rates of HIV testing in all settings over a period of 10 years. Patients with FN samples exited the decision tree under the assumption that they would receive a delayed diagnosis of HIV outside of the TMP setting (i.e., outside the experience of base case patients). Patients with TN samples exited the decision tree.

Patients with TP and FP test results were assumed and modeled in the decision tree to be linked to care or not, and if linked to care, received ART for the model time horizon. They were then modeled in the decision tree to disclose their HIV status to partners or not. Those who did not disclose to partners entered the decision tree branch in which they received HIV partner notification (HPN). Positive partners were entered the decision tree branch in which they received ART for the model time horizon and negative partners (discordant negative) entered the decision tree branch in which they received PrEP for the mean duration of PrEP in Kenya.

Outcomes. We included four outcomes or effectiveness measures as follows: (1) correct diagnostic classification of patients, a factor of both the provider and patient decision to test and the accuracy of the diagnostic testing strategy; (2) HIV case yield; (3) HIV cases (and cases averted); and (4) disability-adjusted life-years (DALYs), the primary outcome in the analysis. The TMP trial was designed to assess the yield of acute and prevalent HIV cases by Xpert RNA testing, as well as linkage and partner outcomes at 6 weeks of follow-up [2]. Data from the trial, the mathematical model, and from the published literature were used to assess test accuracy and HIV case yield (Table 1). The mathematical model allowed for the simulation of the potential prevention impact of the TMP intervention at scale over a 10-year time horizon.[8,10] We adjusted for disability to enable the calculation of DALYs over the 10-year time horizon using disability weights for symptomatic HIV pre-AIDS and HIV/AIDS on ART obtained from the Global Burden of Diseases study (Table 1).[15]

For peer review only - http://bmjopen.bmj.com/site/about/guidelines.xhtml

Perspective. The perspective of the analysis was that of the government of Kenya through the Ministry of Health. Although a significant proportion of the Kenyan population pays for healthcare out of pocket, over 60% of total health expenditure is by the government, which makes it a de facto national payer.[16] As such, the governmental perspective, which includes all formal health sector (medical) costs but excludes all costs that the government would not incur at scale, is important for purposes of government policy making.

Resource use and cost. We divided the cost estimates into two main categories: HIV-testing-related costs and costs related to the management of patients who tested positive for HIV. The costs of HIV testing included the costs of testing supplies and personnel and the costs of HIV-positive patient management included costs of linkage to care, HPN, initiation of ART, maintenance of ART, and PrEP for seronegative partners of patients newly diagnosed through the intervention. The costs of HPN, linkage to care, ART and PrEP were obtained from published studies conducted in Kenya.[17 18]

The costs of testing supplies included the costs of procurement, warranty, maintenance, and electric consumption of the Cepheid GeneXpert HIV-1 POC-NAAT platform. We annuitized the capital cost of the machine and warranty over its estimated useful life and estimated the per-test cost by accounting for throughput of tests per year. We estimated the cost of GeneXpert HIV-1 qual cartridges and the Determine® and First Response® antibody tests on a per-test basis.

To estimate personnel costs, we conducted a primary time motion survey in which we estimated mean service times for pre-test medical consultation, laboratory testing, pre- and post-test counseling (for the POC-NAAT arm), and pharmacy services (survey instrument in supplemental material). The PITC and E-PITC arms of the analysis do not include the personnel costs of pre- and post-test counseling, as pre- and post-test counseling were not a part of the PITC arm in the TMP trial and under the assumption that a future E-PITC activity would proceed without dedicated pre- and post-test counseling services. Time estimates were multiplied by wages for consultants (physicians, clinical officer, or nursing officer), laboratory technicians, pharmacy workers, and counselors.

Currency, price, date, and conversion. Costs were in 2019 $U.S. Cost estimates from years other than 2019 were converted to 2019 costs using the Consumer Price Index for health in Kenya.[19] Costs obtained in local currency units (Kenya shillings) were converted to $US using the Kenya Central Bank rate on July 1st 2019 (1 $US = KSH 101.99).

Discount rate. We used a discount rate of 3% for costs and outcomes as recommended by the 2nd Panel on Cost-Effectiveness in Health and Medicine.[20] This discount rate applies to the annuitization of HIV testing-related capital expenditures as well as costs of ART over the study time horizon. The parameter estimates for costing are presented in Table 1.

Analysis. The base case analysis compared the mean costs, correct classification of patients, HIV cases diagnosed, HIV cases per 10,000 base case patients, and DALYs of PITC, E-PITC, and POC-NAAT. We calculated incremental cost-effectiveness ratios (ICERs) using cost per

For peer review only - http://bmjopen.bmj.com/site/about/guidelines.xhtml

additional correctly classified patient, cost per additional HIV case identified, cost per HIV case averted, and cost per DALY averted. Given three comparators, the aim of the incremental analysis was to identify the most optimal choice by excluding dominated and extended dominated alternatives and identifying the lowest ICER for each comparator by outcome measure. Dominance occurs when one comparator in an incremental cost-effectiveness analysis is less costly and more effective than another comparator. Extended dominance occurs when there are more than two comparators in an incremental cost-effectiveness analysis and one of the comparators is dominated by a linear combination of two other comparators. We also included analyses comparing E-PITC and POC-NAAT independently, pairwise, to PITC. This analysis informs a potential policy choice in which only one of either E-PITC or POC-NAAT were available in a given jurisdiction. As a threshold for cost-effectiveness, we used three times Kenya's per capita gross domestic product (2019), which is $5,450 (World Bank).[21]

We conducted univariate sensitivity analyses to identify the most influential parameters on the results of the analysis of the primary outcome (i.e., DALYs). All parameters were varied across plausible ranges using 95% confidence intervals (CIs) where available and ranges of +/-20% for probabilities and +/-50% for costs when 95% confidence intervals were unavailable (Table 1). We used Monte Carlo simulation to conduct probabilistic sensitivity analyses aimed at assessing overall parameter uncertainty in the model and further testing the robustness of results. We used baseline values as means and estimated standard errors assuming ranges were equivalent to 95% CIs (four times the standard error). We assumed beta distributions for probabilities and disability weights and normal distributions for other parameters. The analysis was performed using TreeAge Pro 2021 and this report conforms to the Consolidated Health Economic Evaluation Reporting Standards (CHEERS) statement.[22]

Results

Base case analysis. Results of the baseline analysis are shown in Table 2. The mean cost of HIV testing per participant (base case patient modeled) under PITC was $0.50, compared to $1.87 for E-PITC, and $29.83 for POC-NAAT. The mean cost of HIV-positive patient management per participant under PITC was $46.74, compared to $171.72 for E-PITC, and $185.08 for POC-NAAT. The total cost of PITC (including both testing and HIV-positive patient management) per participant was therefore $47.24, compared to $173.59 for E-PITC, and $214.91 for POC-NAAT. Therefore, E-PITC increased average costs per participant by $126.34 relative to PITC and POC-NAAT increased costs by $41.30 relative to E-PITC.

Of all base case patients, 25.3% were correctly classified under PITC, compared to 93.7% under E-PITC and 94.9% under POC-NAAT (correct classification was a factor of both the HIV test being performed and the accuracy of the testing strategy). The increase in correct classification comparing E-PITC to PITC (due to scaling up coverage with the same HIV rapid tests from 25.6% to 94.9%) was 68.4%, and the increase in correct classification comparing POC-NAAT to E-PITC (due, predominantly, to diagnosis of acute cases) was 1.1%. The ICER comparing E-PITC to PITC was $185 per additional correctly classified case, and the ICER comparing POC-NAAT to E-PITC was $3,619 per additional correctly classified case.

For peer review only - http://bmjopen.bmj.com/site/about/guidelines.xhtml

PITC had an HIV-positive case yield of 1.25%, compared to 4.60% under E-PITC and 4.65% under POC-NAAT. The increase in HIV-positive case yield comparing E-PITC to PITC was 3.35%, while the increase in HIV-positive case yield comparing POC-NAAT to E-PITC was 0.05%. The ICER comparing E-PITC to PITC was $3,774 per additional HIV case identified, while the ICER comparing POC-NAAT to E-PITC was $83,052 per additional HIV case identified.

In the model, the PITC testing strategy was projected to result in 606 HIV cases per 10,000, compared to 596 HIV cases for the E-PITC testing strategy and 545 HIV cases for POC-NAAT testing strategy. Therefore, E-PITC was projected to avert 10 HIV cases compared to PITC and POC-NAAT was projected to avert 51 cases compared to E-PITC. In the incremental analysis of cases averted, E-PITC was eliminated due to extended dominance (i.e., the cases averted by E-PITC are dominated by a linear combination of the cases averted by PITC and POC-NAAT). The ICER comparing POC-NAAT to PITC was $2.75 per HIV case averted (the ICER comparing POC-NAAT to E-PITC is not calculated in the presence of extended dominance). In an analysis assuming E-PITC as the only available option, the ICER comparing E-PITC to PITC is $12.63 per HIV case averted.

With respect to DALYs, the primary outcome of interest, PITC was associated with a mean of 0.214 DALYs over the 10-year time horizon, compared to 0.176 DALYs for E-PITC and 0.160 DALYs for POC-NAAT. Therefore, E-PITC averted 0.038 DALYs compared to PITC and POC-NAAT averted 0.016 DALYS compared to E-PITC. In the incremental analysis, E-PITC was eliminated by extended dominance given that the DALYs averted by E-PITC are dominated by a linear combination of the DALYs averted by PITC and POC-NAAT. The ICER comparing POC-NAAT to PITC was $3,098 per DALY averted. In a pairwise analysis comparing E-PITC to PITC, an analysis that assumes that E-PITC was the only available alternative to PITC, the ICER was $3,306 per DALY averted.

<u>Sensitivity analysis.</u> The results of the one-way sensitivity analysis are shown as Tornado diagrams in Figure 2. The ICER comparing POC-NAAT to PITC ($/DALY averted) was most sensitive to the disability weight for symptomatic HIV pre-AIDS, the annual cost of ART for patients who tested positive for HIV, the disability weight for HIV/AIDS receiving ART, and the annual per patient cost of ART provided to seropositive partners (Figure 2).

The results of the probabilistic sensitivity analysis are shown in Figure 3 as an incremental cost-effectiveness (ICE) scatterplot and Figure 4 as a cost-effectiveness acceptability curve (CEAC) for the comparison of POC-NAAT and PITC. The ICE scatterplot shows that there was substantial uncertainty as to the impact of POC-NAAT on averting DALYs compared to PITC but near zero uncertainty as to the cost increase of POC-NAAT compared to PITC.

The CEAC shows the probability that PITC and POC-NAAT would be cost-effective in 1,000 runs of Monte Carlo simulation. PITC has a higher probability of being cost-effective than POC-NAAT up to a willingness to pay of just under $2,000 per DALY averted at which point POC-NAAT exhibits a higher probability of being cost-effective. At a willingness to pay of $5,450 per

For peer review only - http://bmjopen.bmj.com/site/about/guidelines.xhtml

DALY averted (the threshold used in the analysis), the distribution of cost-effectiveness in this simulation was 21.9% PITC versus 40.0% POC-NAAT. The probability that PITC would be cost-effective diminishes with increasing willingness to pay, while the probability that POC-NAAT would be cost-effective increases and peaks at 52% at a willingness to pay of approximately $55,000 per DALY averted (Figure 4).

Discussion

We assessed the cost-effectiveness of testing strategies to diagnose only chronic HIV infection (including PITC and expanded antibody testing (E-PITC)) versus a testing strategy to diagnose both acute and chronic HIV infection (i.e., POC-NAAT) in symptomatic adults meeting test criteria in outpatient primary care facilities in Kenya. In terms of correct patient classification, a factor of the decision to test as well as the accuracy of the diagnostic strategy, and HIV-positive patient yield, expanding coverage of rapid antibody testing by testing all patients that meet risk criteria unless they opt out (E-PITC) was more cost-effective than both PITC, the current standard of care, and a testing strategy including HIV-1 RNA testing that was able to diagnose AHI (POC-NAAT). However, in terms of HIV cases averted, a measure of HIV prevention, and DALYs averted, a metric that includes both length and quality of life, POC-NAAT was more cost-effective than both PITC and E-PITC.

With an ICER of $3,306 per DALY averted, and E-PITC eliminated from consideration due to extended dominance, POC-NAAT was cost-effective when considering the traditional threshold of three times GDP per capita: the GDP per capita of Kenya in 2019 was $1,817, suggesting a threshold of $5,450. However, this GDP-based threshold for cost-effectiveness is considered by some to be too high, and programs considered cost-effective are often not implemented in the context of country-specific healthcare spending.[23 24] Some studies suggest thresholds as low as 1% of GDP per capita ($18 in Kenya) in low-income countries when no external funding is available.[25-27] It is also worth noting that HIV has and has had significant amounts of external funding and that higher thresholds are apt in this context.

Previous economic evaluations have demonstrated that testing for AHI is cost-effective in high-risk populations, such as men who have sex with men (MSM) and injection drug users (IDUs) at sufficiently high HIV prevalence (over 0.4%) both in the U.S. and in China.[28] We found no study of the cost-effectiveness of diagnosis for AHI using NAAT in sub-Saharan Africa. However, in countries such as Kenya, where the national HIV prevalence is over 4%, and for patients such as those included the TMP trial (i.e., adults aged 18-39 presenting for outpatient care with symptoms compatible with AHI — the base case patient in this study) the risk of HIV acquisition may be similar to that in the key populations in which previous studies demonstrated that testing for AHI was cost-effective.[29-31]

In one-way sensitivity analysis, the ICER comparing POC-NAAT to PITC was sensitive to disability weights for HIV and costs of ART. The ICER comparing POC-NAAT to PITC increases substantially (less cost-effective) at low disability weights for asymptomatic patients, reflecting the disadvantage of treating relatively well people. The ICER comparing POC-NAAT to PITC was also sensitive to variations in per patient costs of ART both for index patients and partners,

For peer review only - http://bmjopen.bmj.com/site/about/guidelines.xhtml

with higher costs leading to higher ICERs (less cost-effective). This is to be expected because ART is for a lifetime and early diagnosis of HIV as well as the impact of ART to extend life increase the overall cost of ART. Surprisingly, varying the costs of GeneXpert cartridges often considered high in low-income settings did not affect the ICER comparing POC-NAAT to PITC substantially (Figure 2), although low cartridge prices would be desirable from a budget impact (affordability) perspective.

The antibody tests used in the TMP trial, Determine® and First Response®, have near perfect sensitivity and specificity for diagnosis of chronic HIV,[32 33] but are unable to detect AHI. Cepheid's Xpert® HIV-1 Qual platform has high sensitivity and specificity for diagnosis of chronic untreated HIV, with the added benefit of diagnosis of AHI.[34] Despite this ability of NAAT with the Xpert platform to identify AHI, expanded PITC by opt-out in the setting of out-patient clinics (E-PITC) using rapid antibody tests performed well in terms of both accurate classification of patients and yield of HIV-positive patients, as the majority of infections are chronic (see Table 2). Therefore, E-PITC, though not the most cost-effective in terms of cost per DALY averted (the primary outcome), is worth consideration for widespread scale-up a to improve overall accurate classification of patients and yield of HIV positive case relative to the status quo (PITC), despite missing AHI. This point is further reinforced by the results of the probabilistic sensitivity analysis which shows substantial uncertainty in effectiveness (DALYs averted) comparing POC-NAAT and PITC (Figure 3). In this analysis the identification of AHI using POC-NAAT was not as robust in its increase in effectiveness as it was in its increase in cost. If the cost of RNA testing could be reduced (e.g., through reduced cost of GeneXpert cartridges), POC-NAAT would become a more consistently attractive option.

This economic evaluation is generalizable to other, similar countries with high prevalence and populations vulnerable to HIV as assessed by risk scoring at outpatient health facilities. This is because the cost of testing, including the cost of GeneXpert cartridges and antibody tests, is unlikely to vary substantially given that prices are set internationally and countries are exposed to uniform prices, and the cost of other inputs (such as personnel), though variable, were not drivers of cost-effectiveness in the analysis. Most other countries in sub-Saharan Africa that resemble Kenya in this regard face the same issues: how to pay for expanded antibody testing or testing to detect AHI given extreme budget constraints. Given the preventive impact on the HIV epidemic, NAAT testing for AHI in primary care settings should be considered strongly despite higher costs, particularly where dedicated HIV funding is available.

This analysis had a number of strengths. This is the first study in sub-Saharan Africa to consider the cost-effectiveness of NAAT testing for HIV including AHI among symptomatic outpatients. We also included three alternative testing strategy to maximize the policy relevance and potential utility of the study results. One limitation of the analysis was that we conducted the analysis over a time horizon of 10 years and not a lifetime horizon. In using a shorter time horizon, we favored short term certainty in our modeling over long term uncertainty, given that HIV patients now have life expectancies approaching those of the general population. Additionally, while we performed univariate and probabilistic sensitivity analyses to account for

For peer review only - http://bmjopen.bmj.com/site/about/guidelines.xhtml

parameter uncertainty in the decision tree model, we did not account for the additional uncertainty in the number of future HIV cases averted which were obtained from the stochastic network-based model.

As community HIV testing using antibody tests increases in coverage and AHI increases in importance as a driver of HIV incidence, HIV-1 NAAT for AHI at the point of care for patients vulnerable to HIV and meeting AHI screening criteria will increase in importance as a diagnostic mechanism. Our analysis has shown that HIV-1 NAAT for AHI is cost-effective, subject to willingness to pay and the availability of adequate funding. To increase testing coverage and achieve the UNAIDS policy goal of diagnosing 95% of PLWHA, countries like Kenya will face higher costs because the marginal cost of identifying higher percentages of PLWH increases as testing coverage increases. These higher up-front testing costs may be offset, however, by savings related to HIV cases prevented, especially if prompt linkage to care and effective ART limit HIV transmission from index cases. In conclusion, increasing testing coverage for patients vulnerable to HIV at outpatient clinics is cost-effective and will become increasingly so with increasing healthcare budgets and reducing costs of HIV-1 NAAT platforms.

Ethics statement

This study did not receive nor require ethics approval, as it does not involve human & animal participants.

Patient and Public Involvement

Study participants or the public were not involved in the design, or conduct, or reporting, or dissemination plans of our research.

Contributorship

JBB and SM Graham designed the decision model. JBB developed and parameterized the decision model. SM Graham and EJS designed the trial (Tambua Mapema Plus) which formed the main parameterization basis for the decision model. DVT and SM Goodreau developed the stochastic agent-based model which formed the parametrization basis for estimating long-term HIV prevention benefits of HIV testing. CAA led the collection of primary cost data. JBB prepared the draft manuscript. All authors reviewed and contributed to the final manuscript.

Funding

Funding for the Tambua Mapema Trial was provided by the National Institute of Allergy and Infectious Diseases (NIAID), grant R01 AI124968. SMG was also supported by the Robert W. Anderson Endowed Chair in Medicine. We acknowledge Usha Sharma of the NIAID Division of AIDS for the scientific support for the Tambua Mapema Plus study. This work was also supported through the Sub-Saharan African Network for TB/HIV Research Excellence, a Developing Excellence in Leadership, Training and Science (DELTAS) Africa Initiative (grant no.

For peer review only - http://bmjopen.bmj.com/site/about/guidelines.xhtml

DEL-15-006). The DELTAS Africa Initiative is an independent funding scheme of the African Academy of Sciences (AAS)'s Alliance for Accelerating Excellence in Science in Africa and supported by the New Partnership for Africa's Development (NEPAD) Planning and Coordinating Agency with funding from the Wellcome Trust (grant no. 107752/Z/15/Z) and the UK government. Truvada was supplied by Gilead Sciences Inc.

Competing interests

None declared

For peer review only

For peer review only - http://bmjopen.bmj.com/site/about/guidelines.xhtml

References

1. UNAIDS, 2018. UNDETECTABLE = UNTRANSMITTABLE: PUBLIC HEALTH AND HIV VIRAL LOAD SUPPRESSION. Accessed on March 31st 2021 at https://www.unaids.org/sites/default/files/media_asset/undetectable-untransmittable_en.pdf.
2. Understanding Fast-track: Accelerating Action to End the AIDS Epidemic by 2030. Geneva2015.
3. Global AIDS Update 2020. Seizing the Moment: Tackling entrenched inequalities to end epidemics. In: UNAIDS, ed. Geneva: UNAIDS; 2020.
4. Fransen K, de Baetselier I, Rammutla E, et al. Performance of serological and molecular tests within acute HIV infection. *J Clin Virol* 2017;93:81-84. doi: 10.1016/j.jcv.2017.03.010 [published Online First: 2017/03/30]
5. Sanders EJ, Wahome E, Mwangome M, et al. Most adults seek urgent healthcare when acquiring HIV-1 and are frequently treated for malaria in coastal Kenya. *AIDS* 2011;25(9):1219-24. doi: 10.1097/QAD.0b013e3283474ed5 [published Online First: 2011/04/21]
6. Agutu CA, Ngetsa CJ, Price MA, et al. Systematic review of the performance and clinical utility of point of care HIV-1 RNA testing for diagnosis and care. *PLoS One* 2019;14(6):e0218369. doi: 10.1371/journal.pone.0218369 [published Online First: 2019/06/28]
7. Cohen MS, Shaw GM, McMichael AJ, et al. Acute HIV-1 Infection. *N Engl J Med* 2011;364(20):1943-54. doi: 10.1056/NEJMra1011874 [published Online First: 2011/05/20]
8. Graham SM, Agutu C, van der Elst E, et al. A Novel HIV-1 RNA Testing Intervention to Detect Acute and Prevalent HIV Infection in Young Adults and Reduce HIV Transmission in Kenya: Protocol for a Randomized Controlled Trial. *JMIR research protocols* 2020;9(8):e16198. doi: 10.2196/16198 [published Online First: 2020/08/09]
9. Sanders EJ, Agutu C, van der Elst E, et al. Effect of an opt-out point-of-care HIV-1 nucleic acid testing intervention to detect acute and prevalent HIV infection in symptomatic adult outpatients and reduce HIV transmission in Kenya: a randomized controlled trial. *HIV Med* 2021 doi: 10.1111/hiv.13157 [published Online First: 2021/08/26]
10. Modeling the Impact of HIV-1 RNA Testing among Symptomatic Adult Outpatients in Kenya (Forthcoming).
11. Sanders EJ, Wahome E, Powers KA, et al. Targeted screening of at-risk adults for acute HIV-1 infection in sub-Saharan Africa. *AIDS* 2015;29 Suppl 3(0 3):S221-S30. doi: 10.1097/QAD.0000000000000924
12. Kenya Ministry of Health. National AIDS and STI Control Programme. Kenya HIV Estimates, Report 2018. Accessed on 06 29 2020 at https://nacc.or.ke/wp-content/uploads/2018/11/HIV-estimates-report-Kenya-20182.pdf.
13. Agutu CA, Oduor TH, Kombo BK, et al. High patient acceptability but low coverage of provider-initiated HIV testing among adult outpatients with symptoms of acute infectious illness in coastal Kenya. *PloS one* 2021;16(2):e0246444-e44. doi: 10.1371/journal.pone.0246444
14. The Kenya HIV Testing Services Guidelines. Accessed on June 14th 2020 at https://www.fast-trackcities.org/sites/default/files/Kenya%20HIV%20Testing%20Services%20Guidelines%20%282015%29.pdf.

For peer review only - http://bmjopen.bmj.com/site/about/guidelines.xhtml

15. Salomon JA, Haagsma JA, Davis A, et al. Disability weights for the Global Burden of Disease 2013 study. *The Lancet Global Health* 2015;3(11):e712-e23. doi: 10.1016/S2214-109X(15)00069-8
16. Primary health care systems (PRIMASYS): case study from Kenya, abridged version. Geneva: World Health Organization; 2017. Licence: CC BY-NC-SA 3.0 IGO.
17. Sharma M, Smith JA, Farquhar C, et al. Assisted partner notification services are cost-effective for decreasing HIV burden in western Kenya. *AIDS* 2018;32(2):233-41. doi: 10.1097/QAD.0000000000001697
18. Irungu EM, Sharma M, Maronga C, et al. The Incremental Cost of Delivering PrEP as a Bridge to ART for HIV Serodiscordant Couples in Public HIV Care Clinics in Kenya. *AIDS Res Treat* 2019;2019:4170615-15. doi: 10.1155/2019/4170615
19. Kenya National Bureau of Statistics. Kenya Data Portal. Consumer Price Indices (CPI) and Inflation Rates for Kenya. Accessed on 11/03/2020 at https://kenya.opendataforafrica.org/ltziiob/consumer-price-indices-cpi-and-inflation-rates-of-kenya.
20. Neumann PJ, Sanders GD, Russell LB, Siegel JE, and Ganiats TG (Eds). Cost-Effectiveness in Health and Medicine (Second Edition. Oxford University Press, 2017. .
21. World Bank. The World Fact Book. Kenya Per-Capita Gross Domestic in Current Dollars. Accessed at https://data.worldbank.org/indicator/NY.GDP.PCAP.CD on 4th June 2021.
22. Husereau D, Drummond M, Petrou S, et al. Consolidated Health Economic Evaluation Reporting Standards (CHEERS) statement. *BMJ : British Medical Journal* 2013;346:f1049. doi: 10.1136/bmj.f1049
23. Shillcutt SD, Walker DG, Goodman CA, et al. Cost effectiveness in low- and middle-income countries: a review of the debates surrounding decision rules. *Pharmacoeconomics* 2009;27(11):903-17. doi: 10.2165/10899580-000000000-00000
24. Newall AT, Jit M, Hutubessy R. Are Current Cost-Effectiveness Thresholds for Low- and Middle-Income Countries Useful? Examples from the World of Vaccines. *Pharmacoeconomics* 2014;32(6):525-31. doi: 10.1007/s40273-014-0162-x
25. Daroudi R, Akbari Sari A, Nahvijou A, et al. Cost per DALY averted in low, middle- and high-income countries: evidence from the global burden of disease study to estimate the cost-effectiveness thresholds. *Cost Effectiveness and Resource Allocation* 2021;19(1):7. doi: 10.1186/s12962-021-00260-0
26. Ochalek J, Lomas J, Claxton K. Estimating health opportunity costs in low-income and middle-income countries: a novel approach and evidence from cross-country data. *BMJ Global Health* 2018;3(6):e000964. doi: 10.1136/bmjgh-2018-000964
27. Woods B, Revill P, Sculpher M, et al. Country-Level Cost-Effectiveness Thresholds: Initial Estimates and the Need for Further Research. *Value in Health* 2016;19(8):929-35. doi: 10.1016/j.jval.2016.02.017
28. Han X, Xu J, Chu Z, et al. Screening acute HIV infections among Chinese men who have sex with men from voluntary counseling & testing centers. *PLoS One* 2011;6(12):e28792. doi: 10.1371/journal.pone.0028792 [published Online First: 2011/12/24]

For peer review only - http://bmjopen.bmj.com/site/about/guidelines.xhtml

29. Long EF. HIV screening via fourth-generation immunoassay or nucleic acid amplification test in the United States: a cost-effectiveness analysis. *PLoS One* 2011;6(11):e27625. doi: 10.1371/journal.pone.0027625 [published Online First: 2011/11/24]

30. Hoenigl M, Graff-Zivin J, Little SJ. Costs per Diagnosis of Acute HIV Infection in Community-based Screening Strategies: A Comparative Analysis of Four Screening Algorithms. *Clin Infect Dis* 2016;62(4):501-11. doi: 10.1093/cid/civ912 [published Online First: 2015/10/29]

31. Cipriano LE, Zaric GS, Holodniy M, et al. Cost effectiveness of screening strategies for early identification of HIV and HCV infection in injection drug users. *PLoS One* 2012;7(9):e45176. doi: 10.1371/journal.pone.0045176 [published Online First: 2012/10/03]

32. Alere™ Determine™ HIV–1/2 Ag/Ab Combo https://www.fda.gov/media/86959/download.

33. FIRST RESPONSE ®HIV 1-2.O CARD TEST. Rapid Immunochromatographic Card Test for the detection of Antibodies to HIV 1 & 2 in Human Whole Blood/Serum/Plasma. https://www.who.int/diagnostics_laboratory/evaluations/pq-list/hiv-rdts/161220_amended_final_pqpr_0018_010_00_v3.pdf?ua=1.

34. Bwana P, Ageng'o J, Mwau M. Performance and usability of Cepheid GeneXpert HIV-1 qualitative and quantitative assay in Kenya. *PloS one* 2019;14(3):e0213865-e65. doi: 10.1371/journal.pone.0213865

35. Sanders EJ. Effect of a Novel HIV-1 RNA Testing Intevention to Detect Acute and Prevalent HIV Infection in Young Adults and Reduce HIV Transmsission in Kenya: a Randomized Controlled Trial (Forthcoming).

36. Cherutich P, Golden MR, Wamuti B, et al. Assisted partner services for HIV in Kenya: a cluster randomised controlled trial. *The lancet HIV* 2017;4(2):e74-e82. doi: 10.1016/s2352-3018(16)30214-4 [published Online First: 2016/12/04]

37. Government of Kenya. Energy and Petroleum Regulatory Authority. Approved Electricity Charge Rates 2018/2019. (Small COmmercial Customer). Accessed on 08/19/2020 at https://www.epra.go.ke/wp-content/uploads/2018/06/Approved-Tariffs-Aug-2018.pdf.

38. The Global Fund. HIV Viral Load and Early Infant Diagnosis Selection and Procurement Information Tool. Version 2 April 2017. Available at https://www.theglobalfund.org/media/5765/psm_viralloadearlyinfantdiagnosis_content_en.pdf.

39. Walusimbi S, Kwesiga B, Rodrigues R, et al. Cost-effectiveness analysis of microscopic observation drug susceptibility test versus Xpert MTB/Rif test for diagnosis of pulmonary tuberculosis in HIV patients in Uganda. *BMC Health Serv Res* 2016;16(1):563. doi: 10.1186/s12913-016-1804-9 [published Online First: 2016/10/12]

40. National AIDS & STI Control Program, Ministry of Health Kenya. National Quantification report for HIV Commodities for FY 2019/20 to 2021/22, October, 2019.

For peer review only - http://bmjopen.bmj.com/site/about/guidelines.xhtml

599 **Table 1. Model inputs: probabilities, outcomes estimates, resource estimates, and cost**
600 **parameters**

	Base case	Low	High	Reference
**Probabilities**				
Provider-recommended HIV Ab testing	0.256	0.205	0.307	Primary data[35]
Opt-out				
Of physician-recommended Ab test (PITC)	0.000	—	—	Primary data[35]
Of expanded Ab test (E-PITC)	0.051	0.041	0.061	Primary data[35]*
Of RNA test (POC-NAAT)	0.051	0.041	0.061	Primary data[35]
HIV prevalence in Kenya	0.049	0.041	0.210	[12]
Linkage to care				
PITC	0.692	0.554	0.830	Primary data[35]
POC-NAAT/E-PITC	0.892	0.714	1.000	Primary data[35]*
Partner disclosure				
PITC	0.75	0.600	0.900	Primary data[35]
POC-NAAT /E-PITC	0.67	0.536	0.804	Primary data[35]*
Partner positivity				
If true positive	0.230	0.184	0.276	[36]
If false positive	0.049	0.041	0.210	[12¶]
**Disability weights**				
HIV (symptomatic, pre-AIDS)	0.274	0.184	0.377	[15]
HIV/AIDS (receiving ART)	0.078	0.052	0.111	[15]
**Cost inputs**				
GeneXpert				
Electricity consumption (VA)	824	—	—	Cepheid
Cost per unit (KWA) of electricity	$0.14	$0.07	$0.29	[37]
Kenya voltage	240	—	—	Standard
Power factor	0.6	0.48	0.72	Assumption
Machine (GeneXpert IV)	$17,000	$13,600	$20,400	[38]
Warranty	$6,480	$5,184	$7,776	[38]
Cartridge (per test)	$16.80	$13.44	$20.16	[38]
Maintenance (per year)	$1,800	$1,440	$2,160	[38]
Useful life (years)	5	4	6	[39]
Scrap value	$0	—	—	Assumption
Tests per hour	2.5	2.0	3.0	[38]
Annual run time (hours)	2848	2278	3418	Assumption
Determine	$0.82	$0.41	$1.64	[40]
First Response	$0.73	$0.36	$1.46	[40]
Personnel (cadres) by activity				
Clinical consultation				
Medical officers	12.5%	8%	12%	Primary data
Clinical officers	87.5%	72%	100%	Primary data
Lab testing				
Lab technologist	95%	76%	100%	Primary data
Medical lab officer	5%	4%	6%	Primary data
Pharmacy services				

For peer review only

For peer review only - http://bmjopen.bmj.com/site/about/guidelines.xhtml

Clinical officer	5%	4%	6%	Primary data
Pharmacist	10%	8%	12%	Primary data
Pharmacy technologist	85%	68%	100%	Primary data
Time use (minutes)				
Observation arm				
Initial consultation	4	3	5	Primary data
Lab testing	54	31	85	Primary data
Post-lab consultation	4	1	8	Primary data
Pharmacy	2	1	5	Primary data
Intervention arm				
Initial consultation	3	3	5	Primary data
Pre-test counseling	17	14	22	Primary data
Lab testing	68	45	93	Primary data
GeneXpert testing	110	101	144	Primary data
Post-test counseling	3	2	4	Primary data
Post-lab consultation	8	5	14	Primary data
Pharmacy	5	3	7	Primary data
Wages (per hour)				
Medical officers	$1.04	$0.39	$1.56	Primary data[π]
Clinical officers	$0.50	$0.33	$0.75	Primary data[π]
Lab technologist	$0.36	$0.18	$0.54	Primary data[π]
Medical lab officer	$0.47	$0.36	$0.71	Primary data[π]
Pharmacist	$1.08	$0.39	$1.62	Primary data[π]
Pharmacy technologist	$0.35	$0.33	$0.52	Primary data[π]
Counselor	$0.47	$0.33	$0.71	Primary data[π]
Linkage to HIV care[&]	$225.45	$112.73	$338.18	[17]
Antiretroviral therapy				
Initiation	$102.54	$51.27	$153.81	[17]
Maintenance (annual)	$351.10	$175.55	$526.65	[17]
Discounted LYs (10-year time horizon)	8.53			Calculated
Pre-exposure prophylaxis (PrEP) (annual)	$111.29	$83.47	$166.94	[18]
Duration of PrEP (years)	0.315	0.252	0.378	Kenya MOH
Acute partner services (index positive)	$114.39	$57.20	$171.59	[17]

* Assumption: opt out rate, rate pf linkage to care, and rate of partner disclosure in the E-PITC arm are equivalent to rates in the POC-NAAT arm

¶ Assumption: equal to HIV prevalence

π Lower estimate equals starting wage for cadre

& Cost of pre-ART laboratory tests assuming all patients have a CD4 cell count of over 350 cells/μl

For peer review only

Table 2. Mean base case patient costs and outcomes of HIV testing strategies to detect acute and prevalent HIV infection in adult outpatients seeking healthcare for symptoms compatible with acute HIV infection in Kenya

	PITC	E-PITC	POC-NAAT
Costs			
Testing costs	$0.50	$1.87	$29.83
HIV-positive patient management	$46.74	$171.72	$185.08
Total costs	$47.25	$173.59	$214.91
Incremental cost	—	$126.34	$41.32
Testing			
Proportion accurately classified≠	0.2530	0.9374	0.9488
Incremental accurate classification	—	0.6844	0.0114
Cost per additional accurately classified case	—	$184.59	$3,619
HIV positive case yield	0.0125	0.0460	0.0465
Incremental HIV-positive case yield	—	0.0335	0.0005
Cost per additional HIV-positive case identified	—	$3,774	$83,053
HIV prevention			
HIV cases (per 10,000)	606	596	545
HIV cases averted	—	10	51
Cost per HIV case averted	—	Dominated*	$2.75¶
HIV cases averted (reference to PITC)	—	10	61
Cost per HIV case averted (reference to PITC)	—	$12.63	$2.75
Main outcome			
DALYs	0.2140	0.1757	0.1598
DALYs averted	—	0.0382	0.0159
Cost per DALY averted	—	Dominated*	$3,098¶
DALYs averted (reference to PITC)	—	0.0382	0.0541
Cost per DALY averted (reference to PITC)	—	$3,306	$3,098

*E-PITC is excluded from the incremental analysis by extended dominance
¶ICER compares POC-NAAT to PITC
≠A factor of both the decision to test and diagnostic test accuracy

For peer review only

For peer review only - http://bmjopen.bmj.com/site/about/guidelines.xhtml

616 **Figure Titles and Captions**

617

618

619

620

621

622

For peer review only

For peer review only - http://bmjopen.bmj.com/site/about/guidelines.xhtml

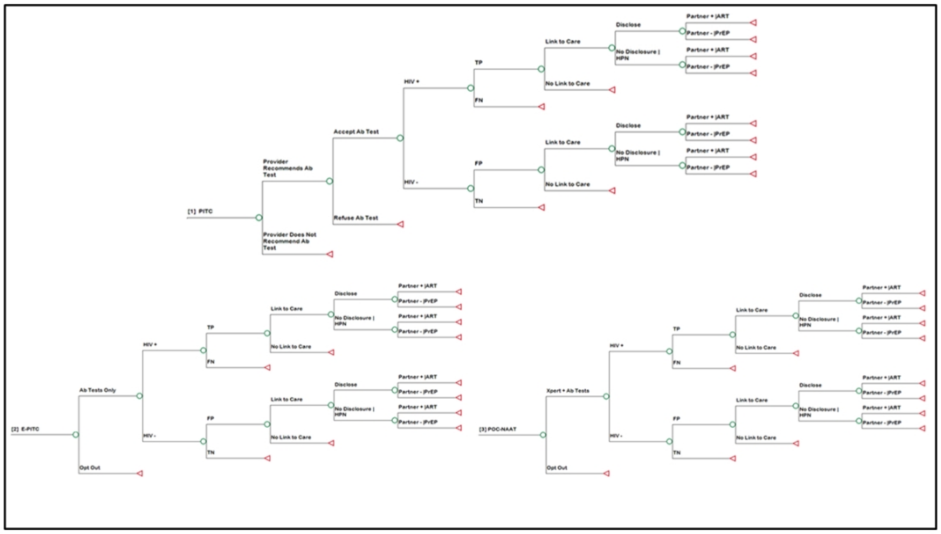

338x190mm (96 x 96 DPI)

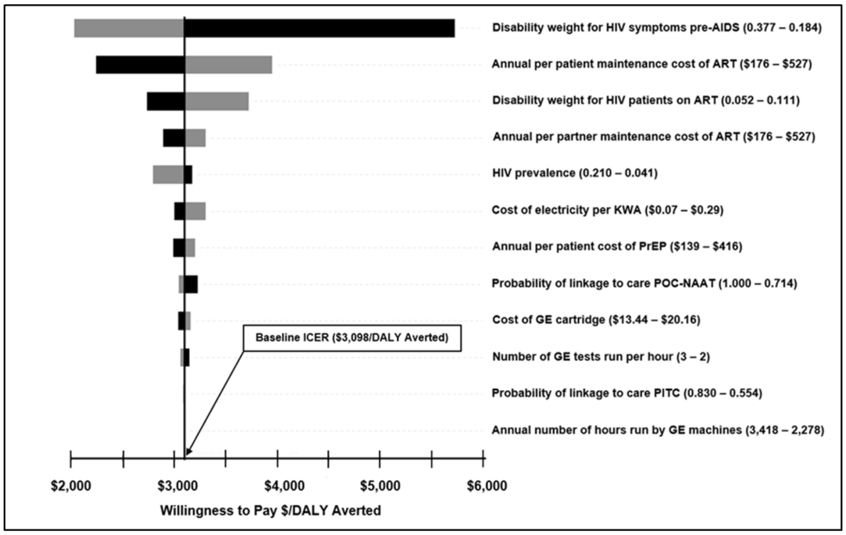

338x190mm (96 x 96 DPI)

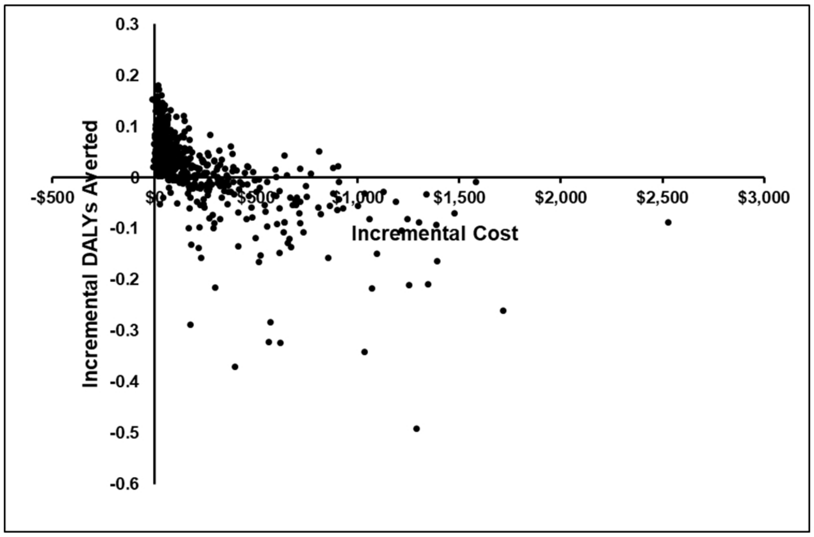

338x190mm (96 x 96 DPI)

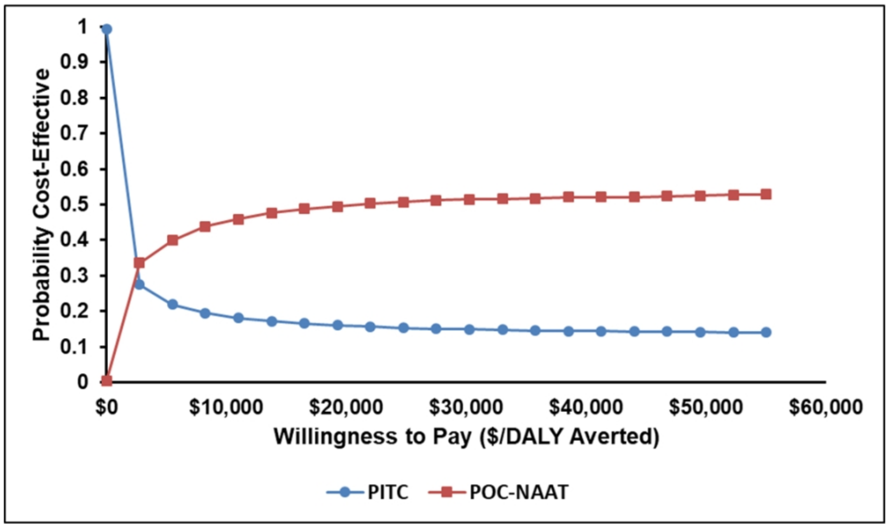

338x190mm (96 x 96 DPI)

CHEERS Checklist

Testing strategies to detect acute and prevalent HIV infection in adult outpatients seeking healthcare for symptoms compatible with acute HIV infection in Kenya: a cost-effectiveness analysis

	Item #	Recommendation	Reported on page #/Line # Comments
**Title and abstract**			
Title	1	Identify the study as an economic evaluation, or use more specific terms such as "cost-effectiveness analysis" and describe the interventions compared.	Page 1, Lines 1,2,3
Abstract	2	Provide a structured summary of objectives, perspective, setting, methods (including study design and inputs), results (including base case and uncertainty analyses), and conclusions.	Page 2, Lines 33 – 57
**Introduction**			
Background and objectives	3	Provide an explicit statement of the broader context for the study.	Page 4, Lines 94 - 107
		Present the study question and its relevance for health policy or practice decisions.	Page 5, Lines 121 - 124
**Methods**			
Target population and subgroups	4	Describe characteristics of the base case population and subgroups analyzed, including why they were chosen.	Page 5, Lines 134 - 139
Setting and location	5	State relevant aspects of the system(s) in which the decision(s) need(s) to be made.	Page 5, Lines 141 - 145
Study perspective	6	Describe the perspective of the study and relate this to the costs being evaluated.	Page 7, Lines 215 - 220
Comparators	7	Describe the interventions or strategies being compared and state why they were chosen.	Pages 5, Lines 147 - 163
Time horizon	8	State the time horizon(s) over which costs and consequences are being evaluated and say why appropriate.	Page 5, Lines 165 - 169
Discount rate	9	Report the choice of discount rate(s) used for costs and outcomes and say why appropriate.	Page 7 Line 251
Choice of health outcomes	10	Describe what outcomes were used as the measure(s) of benefit in the evaluation and their relevance for the type of analysis performed.	Page 6, Lines 202 - 213
Measurement of effectiveness	11a	Single study-based estimates: Describe fully the design features of the single effectiveness study and why the single study was a	NA

For peer review only

		sufficient source of clinical effectiveness data.	
	11b	Synthesis-based estimates: Describe fully the methods used for identification of included studies and synthesis of clinical effectiveness data.	NA
Measurement and valuation of preference-based outcomes	12	If applicable, describe the population and methods used to elicit preferences for outcomes.	NA
Estimating resources and costs	13a	Single study-based economic evaluation: Describe approaches used to estimate resource use associated with the alternative interventions. Describe primary or secondary research methods for valuing each resource item in terms of its unit cost. Describe any adjustments made to approximate to opportunity costs.	NA
	13b	Model-based economic evaluation: Describe approaches and data sources used to estimate resource use associated with model health states. Describe primary or secondary research methods for valuing each resource item in terms of its unit cost. Describe any adjustments made to approximate to opportunity costs.	See table 1, Pages 17 and 18, Lines 606 - 613
Currency, price date, and conversion	14	Report the dates of the estimated resource quantities and unit costs. Describe methods for adjusting estimated unit costs to the year of reported costs if necessary. Describe methods for converting costs into a common currency base and the exchange rate.	Page 7, Lines 246- 249
Choice of model	15	Describe and give reasons for the specific type of decision-analytical model used. Providing a figure to show model structure is strongly recommended	Page 6, Pages 171 - 213; Tree diagram is Figure 1
Assumptions	16	Describe all structural or other assumptions underpinning the decision-analytical model.	Page 6, Lines 190 and 194
Analytic methods	17	Describe all analytical methods supporting the evaluation. This could include methods for dealing with skewed, missing, or censored data; extrapolation methods; methods for pooling data; approaches to validate or make adjustments (such as half cycle corrections) to a model; and	NA

For peer review only

		methods for handling population heterogeneity and uncertainty.	
**Results**			
Study parameters	18	Report the values, ranges, references, and if used, probability distributions for all parameters. Report reasons or sources for distributions used to represent uncertainty where appropriate. Providing a table to show the input values is strongly recommended.	See table 1, Pages 17 and 18, Lines 606 - 613
Incremental costs and outcomes	19	For each intervention, report mean values for the main categories of estimated costs and outcomes of interest, as well as mean differences between the comparator groups. If applicable, report incremental cost-effectiveness ratios.	Table 2, Page 19, Lines 615-620
Characterizing uncertainty	20a	Single study–based economic evaluation: Describe the effects of sampling uncertainty for estimated incremental cost, incremental effectiveness, and incremental cost-effectiveness, together with the impact of methodological assumptions (such as discount rate, study perspective).	NA
	20b	Model-based economic evaluation: Describe the effects on the results of uncertainty for all input parameters, and uncertainty related to the structure of the model and assumptions.	NA
Characterizing heterogeneity	21	If applicable, report differences in costs, outcomes, or cost-effectiveness that can be explained by variations between subgroups of patients with different baseline characteristics or other observed variability in effects that are not reducible by more information.	NA
**Discussion**			
Study findings, limitations, generalizability, and current knowledge	22	Summarize key study findings and describe how they support the conclusions reached. Discuss limitations and the generalizability of the findings and how the findings fit with current knowledge.	Page 10, Lines 354 - 364
**Other**			
Source of funding	23	Describe how the study was funded and the role of the funder in the identification, design, conduct, and reporting of the	Page 12 and 13, Lines 472 - 482

For peer review only

		analysis. Describe other nonmonetary sources of support.	
Conflicts of interest	24	Describe any potential for conflict of interest among study contributors in accordance with journal policy. In the absence of a journal policy, we recommend authors comply with International Committee of Medical Journal Editors' recommendations.	Page 13, Lines 485 - 486

For peer review only

BMJ Open

Testing strategies to detect acute and prevalent HIV infection in adult outpatients seeking healthcare for symptoms compatible with acute HIV infection in Kenya: a cost-effectiveness analysis

Journal:	*BMJ Open*
Manuscript ID	bmjopen-2021-058636.R1
Article Type:	Original research
Date Submitted by the Author:	26-Jul-2022
Complete List of Authors:	Babigumira, Joseph; National University Singapore Saw Swee Hock School of Public Health; National University Health System,<br>Agutu, Clara A.; KEMRI-Wellcome Trust Research Programme<br>Hamilton, Deven T.; University of Washington<br>van der Elst, Elise; KEMRI-Wellcome Trust Research Programme<br>Hassan, Amin; KEMRI-Wellcome Trust Research Programme<br>Gichuru, Evanson; KEMRI-Wellcome Trust Research Programme<br>Mugo, Peter; KEMRI-Wellcome Trust Research Programme, Public health<br>Farquhar, Carey; University of Washington Department of Medicine<br>Ndung'u, Thumbi ; Africa Health Research Institute<br>Sirengo, Martin; National AIDS & STI Control Programme<br>Chege, Wairimu; National Institutes of Health<br>Goodreau, Steven M; University of Washington, Departments of Anthropology and Epidemiology<br>Sanders, Eduard; KEMRI - Wellcome Trust Research Programme; University of Oxford<br>Graham, Susan; KEMRI-Wellcome Trust Research Programme; University of Washington School of Medicine
**Primary Subject Heading**:	Health economics
Secondary Subject Heading:	HIV/AIDS
Keywords:	Health economics < HEALTH SERVICES ADMINISTRATION & MANAGEMENT, Health policy < HEALTH SERVICES ADMINISTRATION & MANAGEMENT, HIV & AIDS < INFECTIOUS DISEASES, HEALTH ECONOMICS

SCHOLARONE™
Manuscripts

I, the Submitting Author has the right to grant and does grant on behalf of all authors of the Work (as defined in the below author licence), an exclusive licence and/or a non-exclusive licence for contributions from authors who are: i) UK Crown employees; ii) where BMJ has agreed a CC-BY licence shall apply, and/or iii) in accordance with the terms applicable for US Federal Government officers or employees acting as part of their official duties; on a worldwide, perpetual, irrevocable, royalty-free basis to BMJ Publishing Group Ltd ("BMJ") its licensees and where the relevant Journal is co-owned by BMJ to the co-owners of the Journal, to publish the Work in this journal and any other BMJ products and to exploit all rights, as set out in our licence.

The Submitting Author accepts and understands that any supply made under these terms is made by BMJ to the Submitting Author unless you are acting as an employee on behalf of your employer or a postgraduate student of an affiliated institution which is paying any applicable article publishing charge ("APC") for Open Access articles. Where the Submitting Author wishes to make the Work available on an Open Access basis (and intends to pay the relevant APC), the terms of reuse of such Open Access shall be governed by a Creative Commons licence – details of these licences and which Creative Commons licence will apply to this Work are set out in our licence referred to above.

Other than as permitted in any relevant BMJ Author's Self Archiving Policies, I confirm this Work has not been accepted for publication elsewhere, is not being considered for publication elsewhere and does not duplicate material already published. I confirm all authors consent to publication of this Work and authorise the granting of this licence.

For peer review only

Testing strategies to detect acute and prevalent HIV infection in adult outpatients seeking healthcare for symptoms compatible with acute HIV infection in Kenya: a cost-effectiveness analysis

Authors

Joseph B. Babigumira[1], Clara A. Agutu[2], Deven T. Hamilton[3], Elise van der Elst[2], Amin Hassan[2], Evanson Gichuru[2]; Peter Mugo[2], Carey Farquhar[4], Thumbi Ndung'u[5], Martin Sirengo[6], Wairimu Chege[7], Steven M. Goodreau[8], Eduard J. Sanders[2,9], Susan M. Graham[2,4]

1. Saw Swee Hock School of Public Health, National University of Singapore and National University Health System, Singapore
2. KEMRI - Wellcome Trust Research Programme, Kilifi, Kenya
3. Center for Studies in Demography & Ecology, University of Washington, Seattle, WA, United States
4. Departments of Medicine, Global Health, and Epidemiology, University of Washington, Seattle, WA, United States
5. Africa Health Research Institute, Durban, South Africa
6. National AIDS & STI Control Programme, Nairobi, Kenya
7. National Institute of Allergy & Infectious Diseases, National Institutes of Health, Rockville, MD, United States
8. Departments of Anthropology and Epidemiology, University of Washington, Seattle, WA, United States
9. University of Oxford, Headington, United Kingdom

Corresponding author

Joseph B. Babigumira
Saw Swee Hock School of Public Health
National University of Singapore
josephbabigumira@gmail.com

For peer review only - http://bmjopen.bmj.com/site/about/guidelines.xhtml

Abstract

Background: Detection of acute and prevalent HIV infection using point-of-care nucleic acid amplification testing (POC-NAAT) among outpatients with symptoms compatible with acute HIV is critical to HIV prevention, but it is not clear if it is cost-effective compared to existing HIV testing strategies.

Methods: We developed and parametrized a decision tree to compare the cost-effectiveness of: (1) provider-initiated testing and counseling (PITC) using rapid tests, the standard of care; (2) scaled-up PITC (SU-PITC) in which all patients were tested with rapid tests unless they opted out; and (3) opt-out testing and counseling using POC-NAAT, which detects both acute and prevalent infection. The model-based analysis used data from the Tambua Mapema Plus randomized controlled trial of a POC-NAAT intervention in Kenya, supplemented with results from a stochastic, agent-based network model of HIV-1 transmission and data from the published literature. The analysis was conducted from the perspective of the Kenyan government using a primary outcome of cost per DALY averted over a 10-year time horizon.

Results: After analyzing the decision-analytic model, the average per patient cost of POC-NAAT was $214.9 compared with $173.6 for SU-PITC and $47.3 for PITC. The mean DALYs accumulated per patient for POC-NAAT were 0.160 compared with 0.176 for SU-PITC and 0.214 for PITC. In the incremental analysis, SU-PITC was eliminated due to extended dominance and the ICER comparing POC-NAAT to PITC was $3,098 per DALY averted. The ICER was sensitive to disability weights for HIV/AIDs and the costs of antiretroviral therapy.

Conclusion: POC-NAAT offered to adult outpatients in Kenya who present for care with symptoms compatible with AHI is cost-effective and should be considered for inclusion as the standard of HIV testing in this population.

For peer review only

For peer review only - http://bmjopen.bmj.com/site/about/guidelines.xhtml

Strengths and limitations of this study

- To our knowledge, this is the first study in sub-Saharan Africa to consider the cost-effectiveness of nucleic acid amplification testing for acute HIV infection in symptomatic outpatients.
- The study included three possible alternatives for acute HIV testing in symptomatic outpatients to maximize local policy relevance.
- The study did not include alternative methods for the diagnosis of acute HIV infection such as rapid antigen-antibody tests.
- The study time horizon was limited to 10 years instead of a lifetime, favoring short term certainty over long term uncertainty.

For peer review only

For peer review only - http://bmjopen.bmj.com/site/about/guidelines.xhtml

Introduction

HIV testing is the gateway to lifelong treatment of HIV-positive individuals with antiretroviral therapy (ART), which prolongs life, reduces morbidity, and optimizes quality of life. HIV testing is also the gateway to HIV prevention through knowledge of HIV status, which empowers HIV-negative individuals to protect themselves, and to treatment as prevention, which effectively renders treated individuals who achieve virologic suppression unable to transmit to others.[1] In 2015, UNAIDS set a target of achieving a 90% rate of diagnosis of people living with HIV (PLWH) by 2020 and a 95% rate of diagnosis of PLWH by 2030, and put both metrics on a "fast track" to ending the AIDS epidemic as a public health threat.[2] However, global progress has not achieved the desired goals: only 81% percent of PLWH knew their status in 2019.[3]

Although Kenya achieved the 90% diagnosis goal for PLWH by the end of 2020[3], reaching the 95% target will be challenging because the current HIV rapid antibody detection tests in use in Kenya are unable to diagnose acute HIV infection (AHI), present during the period before seroconversion.[4] In Kenya, most patients with AHI develop a febrile illness and present to health facilities for urgent care.[5] This provides an opportunity for early diagnosis of AHI at outpatient clinics using newly available nucleic acid amplification testing (NAAT) technologies.[6] Diagnosis of AHI with linkage to care and prompt treatment with ART would lead to important downstream treatment and prevention benefits. Testing for AHI has significant HIV prevention implications because the AHI period is a period of high risk for secondary HIV transmission due to very high viral load.[7] While there are advantages of NAAT in outpatient health facilities in resource-limited settings, these tests remain costly, sometimes up to 20 times the cost of rapid tests on a per-test basis. Consequently, there has been a lack of effectiveness and cost-effectiveness evidence to support the widespread use of NAAT testing in the outpatient setting in Kenya and other sub-Saharan African countries.

To address the effectiveness gap in knowledge, the Tambua Mapema Plus (TMP)[8] study, a modified stepped wedge randomized controlled trial, was conducted. The TMP trial assessed the yield of an opt-out point-of-care (POC) NAAT intervention (POC-NAAT) using Cepheid's Xpert® HIV-1 Qual to diagnose acute and chronic HIV infections in Kenya.[8] In the trial, use of opt-out POC-NAAT resulted in a two-fold greater odds of HIV diagnosis compared to standard of care, i.e. provider-initiated HIV-1 antibody testing.[8] The TMP trial also provided the parameterization basis for the development of a stochastic, agent-based network model to simulate the HIV epidemic in Kenya and quantify the population-level prevention benefits of POC-NAAT testing.[9] The modeling study included a scaled-up provider-initiated testing strategy in which all eligible outpatients were modeled to receive rapid HIV tests unless they opted out. The model projected that using opt-out POC-NAAT was superior to provider-initiated testing or scaled-up HIV rapid testing in terms of both yield of HIV-positives (knowledge of status) and HIV prevention.[9] In this study, we present the results of a cost-effectiveness analysis to address the gap in economic evidence to further support the widespread use of NAAT testing for AHI in the outpatient setting in Kenya and other sub-Saharan African countries.

Methods

For peer review only - http://bmjopen.bmj.com/site/about/guidelines.xhtml

It was not appropriate or possible to involve patients or the public in the design, or conduct, or reporting, or dissemination plans of our research.

Study design. We conducted a synthesis- and model-based cost-effectiveness analysis using costs and outcomes data obtained from the TMP trial[10 11], supplemented by data from a stochastic, agent-based mathematical modeling study[9] and additional data from the published literature. The mathematical model was developed to evaluate the population-level and HIV prevention impact of the TMP intervention and was parameterized using data from the TMP trial.[9] Data from the published literature were used to supplement cost and outcome data obtained from the trial and outcome data obtained from the mathematical model (Table 1).

Target population. The base case population in the analysis included adults 18–39 years, not previously diagnosed with HIV, who presented as outpatients to six public and private primary care health facilities in Kenya with one or more symptoms of AHI. Patients qualified for inclusion in the trial, and consequently in this study, if they had a score of ≥2 on a risk screening score defined by younger age (18–29 years), fever, fatigue, body pains, diarrhea, and sore throat (1 point each), or genital ulcer disease (3 points).[10 12]

Setting. The study setting and decision-making context was peri-urban coastal Kenya (Mombasa and Kilifi counties), an area with busy nightlife, sex work, and tourism (estimated HIV prevalence of 5.6% and 2.3% for Mombasa and Kilifi counties respectively in 2018.[13]) Therefore, insights from this study may also have decision-making relevance in urban and peri-urban settings in Kenya and other, similar low-income, high HIV burden countries.

Comparators. We compared three HIV testing strategies as follows:

1. Provider-initiated HIV-1 testing and counseling (PITC), the standard of current care in Kenya. Under PITC, providers ordered rapid HIV-1 antibody testing at their discretion as a diagnostic aid. This comparator is identical to the observation arm in the TMP trial, in which only 352 (25.6%) of 1374 enrolled participants were tested for HIV and 13 (0.9%) of 1374 enrolled participants were newly diagnosed with HIV.[14]
2. Scaled-up PITC (SU-PITC), in which providers were modeled to order rapid HIV-1 antibody testing for all patients unless they opted out of HIV testing. This comparator was included as a potentially plausible policy alternative for testing among this group of outpatients, given that it excludes relatively costly POC-NAAT.
3. POC-NAAT testing and counseling using point-of-care Xpert® HIV-1 Qual, as performed under the intervention arm of the TMP trial. Under this POC-NAAT approach, providers ordered NAAT testing for all included patients except those who were eligible but opted out of the study. Patients who tested positive by POC-NAAT received rapid HIV-1 antibody testing to distinguish between acute and prevalent HIV infection. This comparator is identical to the intervention arm in the TMP trial, in which all 1,500 participants were tested for HIV, and 37 (2.5%) were newly diagnosed with HIV, of whom 2 were AHI cases.[11]

Time horizon. The analysis was conducted over a time horizon of 10 years, consistent with the mathematical model[9], and thereby included the 10-year costs of ART for individuals that tested

For peer review only - http://bmjopen.bmj.com/site/about/guidelines.xhtml

positive for HIV and costs of pre-exposure prophylaxis (PrEP) for HIV-negative partners of newly diagnosed index patients that were based on the mean duration of PrEP use in Kenya (Table 1).

Decision analytic model. We developed a decision-analytic model (decision tree) to assess the cost-effectiveness of the three different HIV testing strategies. Figures 1-3 show the decision tree branches proceeding from the decision node representing the choice between PITC (Figure 1), SU-PITC (Figure 2), and POC-NAAT (Figure 3). The data used to parameterize the model, including ranges used for sensitivity analysis, are shown in detail in Table 1. In PITC, base-case patients received a Determine® HIV antibody test at the recommendation and discretion of the provider unless they refused HIV testing. In SU-PITC, all patients received a Determine® HIV antibody test unless they opted out. Patients who tested positive with Determine® in either scenario received confirmatory testing with the First Response® HIV antibody test. In POC-NAAT, patients who had positive HIV-1 RNA Qual results received HIV rapid testing with the two different antibody tests in parallel, to distinguish acute from prevalent HIV infection. In each study arm, HIV rapid tests were repeated for confirmation of diagnosis prior to ART initiation, in accordance with Kenyan guidelines.[15]

The decision tree then divided patients into HIV-positive and HIV-negative based on the prevalence of HIV in Kenya.[13] The decision tree assessed the accuracy of testing strategies by dividing patient test results into true positive (TP), false negative (FN), false positive (FP) and true negative (TN), based on estimates from the mathematical model.[9] The decision tree simulated the experience of base-case patients and their likelihood of ending up in the four diagnostic accuracy groups, given modeled HIV transmission risk, rates of HIV rapid testing in the outpatient setting, and background rates of HIV testing in all settings over a period of 10 years. Patients with FN samples exited the decision tree under the assumption that they would receive a delayed diagnosis of HIV outside of the TMP setting (i.e., outside the experience of base case patients). Patients with TN samples exited the decision tree.

Patients with TP and FP test results were assumed and modeled in the decision tree to be linked to care or not, and if linked to care, received ART for the model time horizon. They were then modeled in the decision tree to disclose their HIV status to partners or not. Those who did not disclose to partners entered the decision tree branch in which they received HIV partner notification (HPN). Positive partners entered the decision tree branch in which they received ART for the model time horizon and negative partners (discordant negative) entered the decision tree branch in which they received PrEP for the mean duration of PrEP in Kenya.

Outcomes. In addition to the estimation of the mean costs of the comparators over the time horizon of the study, we included four outcomes or effectiveness measures as follows: (1) correct diagnostic classification of patients, a factor of both the provider and patient decision to test and the accuracy of the diagnostic testing strategy; (2) HIV case yield; (3) HIV cases (and cases averted); and (4) disability-adjusted life-years (DALYs), the primary outcome in the analysis. The TMP trial was designed to assess the yield of acute and prevalent HIV cases by Xpert RNA testing, as well as linkage and partner outcomes at 6 weeks of follow-up[10]. Data from

For peer review only - http://bmjopen.bmj.com/site/about/guidelines.xhtml

the trial, the mathematical model, and from the published literature were used to assess test accuracy and HIV case yield (Table 1). The mathematical model allowed for the simulation of the potential prevention impact of the TMP intervention at scale over a 10-year time horizon.[9]

Perspective. The perspective of the analysis was that of the government of Kenya through the Ministry of Health. Although a significant proportion of the Kenyan population pays for healthcare out of pocket, over 60% of total health expenditure is by the government, which makes it a de facto national payer.[16] As such, the governmental perspective, which includes all formal health sector (medical) costs but excludes all costs that the government would not incur at scale, is important for purposes of government policy making.

Resource use and cost. We divided the cost estimates into two main categories: HIV-testing-related costs and costs related to the management of patients who tested positive for HIV. The costs of HIV testing included the costs of testing supplies and personnel and the costs of HIV-positive patient management included costs of linkage to care, HPN, initiation of ART, maintenance of ART, and PrEP for seronegative partners of patients newly diagnosed through the intervention. The costs of HPN, linkage to care, ART and PrEP were obtained from published studies conducted in Kenya.[17 18]

The costs of testing supplies included the costs of procurement, warranty, maintenance, and electric consumption of the Cepheid GeneXpert HIV-1 POC-NAAT platform. We annuitized the capital cost of the machine and warranty over its estimated useful life and estimated the per-test cost by accounting for throughput of tests per year. We estimated the cost of GeneXpert HIV-1 qual cartridges and the Determine® and First Response® antibody tests on a per-test basis.

To estimate personnel costs, we conducted a primary time motion survey in which we estimated mean service times for pre-test medical consultation, laboratory testing, pre- and post-test counseling (for the POC-NAAT arm), and pharmacy services (survey instrument in supplemental material). The PITC and SU-PITC arms of the analysis do not include the personnel costs of pre- and post-test counseling, as pre- and post-test counseling were not a part of the PITC arm in the TMP trial and under the assumption that a future SU-PITC activity would proceed without dedicated pre- and post-test counseling services. Time estimates were multiplied by wages for consultants (physicians, clinical officer, or nursing officer), laboratory technicians, pharmacy workers, and counselors.

Currency, price, date, and conversion. Costs were in 2019 $U.S. Cost estimates from years other than 2019 were converted to 2019 costs using the Consumer Price Index for health in Kenya.[19] Costs obtained in local currency units (Kenya shillings) were converted to $US using the Kenya Central Bank rate on July 1st 2019 (1 $US = KSH 101.99).

Discount rate. We used a discount rate of 3% for costs and outcomes as recommended by the 2nd Panel on Cost-Effectiveness in Health and Medicine.[20] This discount rate applies to the annuitization of HIV testing-related capital expenditures as well as costs of ART over the study time horizon. The parameter estimates for costing are presented in Table 1.

For peer review only - http://bmjopen.bmj.com/site/about/guidelines.xhtml

Analysis. The base case analysis compared the mean costs, correct classification of patients, HIV cases diagnosed, HIV cases per 10,000 base case patients, and DALYs of PITC, SU-PITC, and POC-NAAT. We adjusted for disability to enable the calculation of DALYs over the 10-year time horizon using disability weights for symptomatic HIV pre-AIDS and HIV/AIDS on ART obtained from the Global Burden of Diseases study (Table 1).[21] We stratified the population of patients by AIDS status (i.e. pre-AIDS and AIDS) and adjusted their years of life lived with pre-AIDS and AIDS by their associated disability weights and added the years of life lost due to premature mortality by subtracting mean age at death from the life expectancy in Kenya. This stratification method and published disability weights were used because we lacked patient-specific disability weights (obtained from person trade-off studies).

We calculated incremental cost-effectiveness ratios (ICERs) using cost per additional correctly classified patient, cost per additional HIV case identified, cost per HIV case averted, and cost per DALY averted. ICERs were calculated by dividing the difference in costs between pairs of comparators by the difference in outcomes for the same comparators. Given three comparators, the aim of the incremental analysis was to identify the most optimal choice by excluding dominated and extended dominated alternatives and identifying the lowest ICER for each comparator by outcome measure. Dominance occurs when one comparator in an incremental cost-effectiveness analysis is less costly and more effective than another comparator. Extended dominance occurs when there are more than two comparators in an incremental cost-effectiveness analysis and one of the comparators is dominated by a linear combination of two other comparators. We also included analyses comparing SU-PITC and POC-NAAT independently, pairwise, to PITC. This analysis informs a potential policy choice in which only one of either SU-PITC or POC-NAAT were available in a given jurisdiction. As a threshold for cost-effectiveness, we used three times Kenya's per capita gross domestic product (2019), which is $5,450 (World Bank).[22]

We conducted univariate sensitivity analyses to identify the most influential parameters on the results of the analysis of the primary outcome (i.e., DALYs). All parameters were varied across plausible ranges using 95% confidence intervals (CIs) where available and ranges of +/-20% for probabilities and +/-50% for costs when 95% confidence intervals were unavailable (Table 1). The use of these arbitrary value ranges in the absence of confidence intervals is often used in the literature[23 24] and reflects the higher uncertainty observed in cost estimates compared to estimates of other parameters. We used Monte Carlo simulation to conduct probabilistic sensitivity analyses aimed at assessing overall parameter uncertainty in the model and further testing the robustness of results. We used baseline values as means and estimated standard errors assuming ranges were equivalent to 95% CIs (four times the standard error). We assumed beta distributions for probabilities and disability weights and normal distributions for other parameters. The analysis was performed using TreeAge Pro 2021 and this report conforms to the Consolidated Health Economic Evaluation Reporting Standards (CHEERS) statement.[25]

Results

For peer review only - http://bmjopen.bmj.com/site/about/guidelines.xhtml

Base case analysis. Results of the baseline analysis are shown in Table 2. The mean cost of HIV testing per participant (base case patient modeled) under PITC was $0.50, compared to $1.87 for SU-PITC, and $29.83 for POC-NAAT. The mean cost of HIV-positive patient management per participant under PITC was $46.74, compared to $171.72 for SU-PITC, and $185.08 for POC-NAAT. The total cost of PITC (including both testing and HIV-positive patient management) per participant was therefore $47.24, compared to $173.59 for SU-PITC, and $214.91 for POC-NAAT. Therefore, SU-PITC increased average costs per participant by $126.34 relative to PITC and POC-NAAT increased costs by $41.30 relative to SU-PITC.

Of all base case patients, 25.3% were correctly classified under PITC, compared to 93.7% under SU-PITC and 94.9% under POC-NAAT (correct classification was a factor of both the HIV test being performed and the accuracy of the testing strategy). The increase in correct classification comparing SU-PITC to PITC (due to scaling up coverage with the same HIV rapid tests from 25.6% to 94.9%) was 68.4%, and the increase in correct classification comparing POC-NAAT to SU-PITC (due, predominantly, to diagnosis of acute cases) was 1.1%. The ICER comparing SU-PITC to PITC was $185 per additional correctly classified case, and the ICER comparing POC-NAAT to SU-PITC was $3,619 per additional correctly classified case.

PITC had an HIV-positive case yield of 1.25%, compared to 4.60% under SU-PITC and 4.65% under POC-NAAT. The increase in HIV-positive case yield comparing SU-PITC to PITC was 3.35%, while the increase in HIV-positive case yield comparing POC-NAAT to SU-PITC was 0.05%. The ICER comparing SU-PITC to PITC was $3,774 per additional HIV case identified, while the ICER comparing POC-NAAT to SU-PITC was $83,052 per additional HIV case identified.

In the model, the PITC testing strategy was projected to result in 606 HIV cases per 10,000, compared to 596 HIV cases for the SU-PITC testing strategy and 545 HIV cases for POC-NAAT testing strategy. Therefore, SU-PITC was projected to avert 10 HIV cases compared to PITC and POC-NAAT was projected to avert 51 cases compared to SU-PITC. In the incremental analysis of cases averted, SU-PITC was eliminated due to extended dominance (i.e., the cases averted by SU-PITC are dominated by a linear combination of the cases averted by PITC and POC-NAAT). The ICER comparing POC-NAAT to PITC was $2.75 per HIV case averted (the ICER comparing POC-NAAT to SU-PITC is not calculated in the presence of extended dominance). In an analysis assuming SU-PITC as the only available option, the ICER comparing SU-PITC to PITC is $12.63 per HIV case averted.

With respect to DALYs, the primary outcome of interest, PITC was associated with a mean of 0.214 DALYs over the 10-year time horizon, compared to 0.176 DALYs for SU-PITC and 0.160 DALYs for POC-NAAT. Therefore, SU-PITC averted 0.038 DALYs compared to PITC and POC-NAAT averted 0.016 DALYS compared to SU-PITC. In the incremental analysis, SU-PITC was eliminated by extended dominance given that the DALYs averted by SU-PITC are dominated by a linear combination of the DALYs averted by PITC and POC-NAAT. The ICER comparing POC-NAAT to PITC was $3,098 per DALY averted. In a pairwise analysis comparing SU-PITC

For peer review only - http://bmjopen.bmj.com/site/about/guidelines.xhtml

to PITC, an analysis that assumes that SU-PITC was the only available alternative to PITC, the ICER was $3,306 per DALY averted.

Sensitivity analysis. The results of the one-way sensitivity analysis are shown as Tornado diagrams in Figure 4. The ICER comparing POC-NAAT to PITC ($/DALY averted) was most sensitive to the disability weight for symptomatic HIV pre-AIDS, the annual cost of ART for patients who tested positive for HIV, the disability weight for HIV/AIDS receiving ART, and the annual per patient cost of ART provided to seropositive partners (Figure 4).

The results of the probabilistic sensitivity analysis are shown in Figure 5 as an incremental cost-effectiveness (ICE) scatterplot and Figure 6 as a cost-effectiveness acceptability curve (CEAC) for the comparison of POC-NAAT and PITC. The ICE scatterplot shows that there was substantial uncertainty as to the impact of POC-NAAT on averting DALYs compared to PITC but near zero uncertainty as to the cost increase of POC-NAAT compared to PITC.

The CEAC shows the probability that PITC and POC-NAAT would be cost-effective in 1,000 runs of Monte Carlo simulation. PITC has a higher probability of being cost-effective than POC-NAAT up to a willingness to pay of just under $2,000 per DALY averted at which point POC-NAAT exhibits a higher probability of being cost-effective. At a willingness to pay of $5,450 per DALY averted (the threshold used in the analysis), the distribution of cost-effectiveness in this simulation was 21.9% PITC versus 40.0% POC-NAAT. The probability that PITC would be cost-effective diminishes with increasing willingness to pay, while the probability that POC-NAAT would be cost-effective increases and peaks at 52% at a willingness to pay of approximately $55,000 per DALY averted (Figure 6).

Discussion

We assessed the cost-effectiveness of testing strategies to diagnose only chronic HIV infection (including PITC and scaled-up antibody testing (SU-PITC)) versus a testing strategy to diagnose both acute and chronic HIV infection (i.e., POC-NAAT) in symptomatic adults meeting test criteria in outpatient primary care facilities in Kenya. In terms of correct patient classification, a factor of the decision to test as well as the accuracy of the diagnostic strategy, and HIV-positive patient yield, expanding coverage of rapid antibody testing by testing all patients that meet risk criteria unless they opt out (SU-PITC) was more cost-effective than both PITC, the current standard of care, and a testing strategy including HIV-1 RNA testing that was able to diagnose AHI (POC-NAAT). However, in terms of HIV cases averted, a measure of HIV prevention, and DALYs averted, a metric that includes both length and quality of life, POC-NAAT was more cost-effective than both PITC and SU-PITC.

With an ICER of $3,306 per DALY averted, and SU-PITC eliminated from consideration due to extended dominance, POC-NAAT was cost-effective when considering the traditional threshold of three times GDP per capita: the GDP per capita of Kenya in 2019 was $1,817, suggesting a threshold of $5,450. However, this GDP-based threshold for cost-effectiveness is considered by some to be too high, and programs considered cost-effective are often not implemented in the context of country-specific healthcare spending.[26 27] Some studies suggest thresholds as low as

For peer review only - http://bmjopen.bmj.com/site/about/guidelines.xhtml

1% of GDP per capita ($18 in Kenya) in low-income countries when no external funding is available.[28-30] It is also worth noting that HIV has and has had significant amounts of external funding and that higher thresholds are apt in this context.

Previous economic evaluations have demonstrated that testing for AHI is cost-effective in high-risk populations, such as men who have sex with men (MSM) and injection drug users (IDUs) at sufficiently high HIV prevalence (over 0.4%) both in the U.S. and in China.[31] We found no study of the cost-effectiveness of diagnosis for AHI using NAAT in sub-Saharan Africa. However, in countries such as Kenya, where the national HIV prevalence is over 4%, and for patients such as those included the TMP trial (i.e., adults aged 18-39 presenting for outpatient care with symptoms compatible with AHI — the base case patient in this study) the risk of HIV acquisition may be similar to that in the key populations in which previous studies demonstrated that testing for AHI was cost-effective.[32-34]

In one-way sensitivity analysis, the ICER comparing POC-NAAT to PITC was sensitive to disability weights for HIV and costs of ART. The ICER comparing POC-NAAT to PITC increases substantially (less cost-effective) at low disability weights for asymptomatic patients, reflecting the disadvantage of treating relatively well people. The ICER comparing POC-NAAT to PITC was also sensitive to variations in per patient costs of ART both for index patients and partners, with higher costs leading to higher ICERs (less cost-effective). This is to be expected because ART is for a lifetime and early diagnosis of HIV as well as the impact of ART to extend life increase the overall cost of ART. Surprisingly, varying the costs of GeneXpert cartridges often considered high in low-income settings did not affect the ICER comparing POC-NAAT to PITC substantially (Figure 4), although low cartridge prices would be desirable from a budget impact (affordability) perspective.

The antibody tests used in the TMP trial, Determine® and First Response®, have near perfect sensitivity and specificity for diagnosis of chronic HIV,[35 36] but are unable to detect AHI. Cepheid's Xpert® HIV-1 Qual platform has high sensitivity and specificity for diagnosis of chronic untreated HIV, with the added benefit of diagnosis of AHI.[37] Despite this ability of NAAT with the Xpert platform to identify AHI, scaled-up PITC by opt-out in the setting of out-patient clinics (SU-PITC) using rapid antibody tests performed well in terms of both accurate classification of patients and yield of HIV-positive patients, as the majority of infections are chronic (see Table 2). Therefore, SU-PITC, though not the most cost-effective in terms of cost per DALY averted (the primary outcome), is worth consideration for widespread scale-up a to improve overall accurate classification of patients and yield of HIV positive case relative to the status quo (PITC), despite missing AHI. This point is further reinforced by the results of the probabilistic sensitivity analysis which shows substantial uncertainty in effectiveness (DALYs averted) comparing POC-NAAT and PITC (Figure 5). In this analysis the identification of AHI using POC-NAAT was not as robust in its increase in effectiveness as it was in its increase in cost. If the cost of RNA testing could be reduced (e.g., through reduced cost of GeneXpert cartridges), POC-NAAT would become a more consistently attractive option.

For peer review only - http://bmjopen.bmj.com/site/about/guidelines.xhtml

This economic evaluation is generalizable to other, similar countries with high prevalence and populations vulnerable to HIV as assessed by risk scoring at outpatient health facilities. This is because the cost of testing, including the cost of GeneXpert cartridges and antibody tests, is unlikely to vary substantially given that prices are set internationally and countries are exposed to uniform prices, and the cost of other inputs (such as personnel), though variable, were not drivers of cost-effectiveness in the analysis. Most other countries in sub-Saharan Africa that resemble Kenya in this regard face the same issues: how to pay for scaled-up antibody testing or testing to detect AHI given extreme budget constraints. Given the preventive impact on the HIV epidemic, NAAT testing for AHI in primary care settings should be considered strongly despite higher costs, particularly where dedicated HIV funding is available.

This analysis had a number of strengths. This is the first study in sub-Saharan Africa to consider the cost-effectiveness of NAAT testing for HIV including AHI among symptomatic outpatients. We also included three alternative testing strategy to maximize the policy relevance and potential utility of the study results. One limitation of the analysis was that we conducted the analysis over a time horizon of 10 years and not a lifetime horizon. In using a shorter time horizon, we favored short term certainty in our modeling over long term uncertainty, given that HIV patients now have life expectancies approaching those of the general population. Additionally, while we performed univariate and probabilistic sensitivity analyses to account for parameter uncertainty in the decision tree model, we did not account for the additional uncertainty in the number of future HIV cases averted which were obtained from the stochastic network-based model. Lastly, alternative methods to diagnose AHI (e.g., rapid antigen-antibody tests) were not considered in this cost-effectiveness analysis.

As community HIV testing using antibody tests increases in coverage and AHI increases in importance as a driver of HIV incidence, HIV-1 NAAT for AHI at the point of care for patients vulnerable to HIV and meeting AHI screening criteria will increase in importance as a diagnostic mechanism. Our analysis has shown that HIV-1 NAAT for AHI is cost-effective, subject to willingness to pay and the availability of adequate funding. To increase testing coverage and achieve the UNAIDS policy goal of diagnosing 95% of PLWHA, countries like Kenya will face higher costs because the marginal cost of identifying higher percentages of PLWH increases as testing coverage increases. These higher up-front testing costs may be offset, however, by savings related to HIV cases prevented, especially if prompt linkage to care and effective ART limit HIV transmission from index cases. In conclusion, increasing testing coverage for patients vulnerable to HIV at outpatient clinics is cost-effective and will become increasingly so with increasing healthcare budgets and reducing costs of HIV-1 NAAT platforms.

Ethics statement

This study did not receive nor require ethics approval, as it does not involve human & animal participants.

Patient and Public Involvement

For peer review only - http://bmjopen.bmj.com/site/about/guidelines.xhtml

For peer review only

Study participants or the public were not involved in the design, or conduct, or reporting, or dissemination plans of our research.

Contributorship

JBB: study conception and design; design of decision-analytic model; analysis of primary data; development and parameterization of the decision-analytic model; preparation of the first draft of the manuscript; and review and revision of drafts of the manuscript. CAA: leadership, collection, and analysis of primary data; and review and revision of drafts of the manuscript. DVT: development of stochastic agent-based mathematical model for parameterization of the decision-analytic model; parameterization of decision model; and review and revision of drafts of the manuscript. EvdE: primary data collection; and review and revision of drafts of the manuscript. AH: primary data collection; and review and revision of drafts of the manuscript. EG: primary data collection; and review and revision of drafts of the manuscript. PM: primary data collection; and review and revision of drafts of the manuscript. CF: study conception and design; and review and revision of drafts of the manuscript. TN: primary data collection; and review and revision of drafts of the manuscript. MS: primary data collection; and review and revision of drafts of the manuscript. WC: primary data collection; and review and revision of drafts of the manuscript. SM Goodreau: development of stochastic agent-based mathematical model for parameterization of the decision-analytic model; and review and revision of drafts of the manuscript. EJS: study conception and design; conduct of trial (Tambua Mapema Plus) which formed the main parameterization basis for the decision-analytic model; parameterization of the decision-analytic model; and review and revision of drafts of the manuscript. SM Graham: study conception and design, design of decision-analytic model; parametrization of decision-analytic model, conduct of trial (Tambua Mapema Plus) which formed the main parameterization basis for the decision-analytic model; and review and revision of drafts of the manuscript. All authors: review, revision, and approval of the final draft of the manuscript.

Funding

Funding for the Tambua Mapema Trial was provided by the National Institute of Allergy and Infectious Diseases (NIAID), grant R01 AI124968. SMG was also supported by the Robert W. Anderson Endowed Chair in Medicine. We acknowledge Usha Sharma of the NIAID Division of AIDS for the scientific support for the Tambua Mapema Plus study. This work was also supported through the Sub-Saharan African Network for TB/HIV Research Excellence, a Developing Excellence in Leadership, Training and Science (DELTAS) Africa Initiative (grant no. DEL-15-006). The DELTAS Africa Initiative is an independent funding scheme of the African Academy of Sciences (AAS)'s Alliance for Accelerating Excellence in Science in Africa and supported by the New Partnership for Africa's Development (NEPAD) Planning and Coordinating Agency with funding from the Wellcome Trust (grant no. 107752/Z/15/Z) and the UK government. Truvada was supplied by Gilead Sciences Inc.

Competing interests

For peer review only - http://bmjopen.bmj.com/site/about/guidelines.xhtml

None declared

Data availability statement

Data available upon reasonable request.

Figure captions

Figure 1. Decision tree branch showing the consequences of Provider-Initiated Testing and Counselling (PITC). The decision tree branch follows a decision node. Ab – Antibody, TP – True Positive, FN – False Negative; FP – False Positive, TN – True Negative; HPN – HIV Partner Notification, ART – Antiretroviral Therapy, PrEP – Pre-Exposure Prophylaxis.

Figure 2. Decision tree branch showing the consequences of Scaled-Up Provider-Initiated Testing and Counselling (SU-PITC). The decision tree branch follows a decision node. Ab – Antibody, TP – True Positive, FN – False Negative; FP – False Positive, TN – True Negative; HPN – HIV Partner Notification, ART – Antiretroviral Therapy, PrEP – Pre-Exposure Prophylaxis.

Figure 3. Decision tree branch showing the consequences of Point-of-Care Nucleic Acid Amplification Testing (POC-NAAT). The decision tree branch follows a decision node. Ab – Antibody, TP – True Positive, FN – False Negative; FP – False Positive, TN – True Negative; HPN – HIV Partner Notification, ART – Antiretroviral Therapy, PrEP – Pre-Exposure Prophylaxis.

Figure 4. Tornado diagram of univariate sensitivity analysis showing the model parameters with the most influence on the cost per DALY averted comparing point-of-care nucleic acid amplification testing (POC-NAAT) and provider-initiated testing and counseling (PITC). ART – Antiretroviral Therapy, KWA – Kilowatt Hour, GE – GeneXpert, DALY – Disability-Adjusted Life-Year.

Figure 5. Incremental cost-effectiveness scatterplot of probabilistic sensitivity analysis showing the distribution of cost-effectiveness pairs on the cost-effectiveness plane. DALYs – Disability-Adjusted Life-Years

Figure 6. Cost-effectiveness acceptability curve showing the probability that point-of-care nucleic acid amplification testing (POC-NAAT) is cost-effective compared to provider-initiated testing and counseling (PITC) at varying levels of willingness to pay for a disability-adjusted life year (DALY) averted. DALY – Disability-Adjusted Life-Year

For peer review only - http://bmjopen.bmj.com/site/about/guidelines.xhtml

References

1. UNAIDS, 2018. UNDETECTABLE = UNTRANSMITTABLE: PUBLIC HEALTH AND HIV VIRAL LOAD SUPPRESSION. Accessed on March 31st 2021 at https://www.unaids.org/sites/default/files/media_asset/undetectable-untransmittable_en.pdf.
2. Understanding Fast-track: Accelerating Action to End the AIDS Epidemic by 2030. Geneva2015.
3. Global AIDS Update 2020. Seizing the Moment: Tackling entrenched inequalities to end epidemics. In: UNAIDS, ed. Geneva: UNAIDS; 2020.
4. Fransen K, de Baetselier I, Rammutla E, et al. Performance of serological and molecular tests within acute HIV infection. *J Clin Virol* 2017;93:81-84. doi: 10.1016/j.jcv.2017.03.010 [published Online First: 2017/03/30]
5. Sanders EJ, Wahome E, Mwangome M, et al. Most adults seek urgent healthcare when acquiring HIV-1 and are frequently treated for malaria in coastal Kenya. *AIDS* 2011;25(9):1219-24. doi: 10.1097/QAD.0b013e3283474ed5 [published Online First: 2011/04/21]
6. Agutu CA, Ngetsa CJ, Price MA, et al. Systematic review of the performance and clinical utility of point of care HIV-1 RNA testing for diagnosis and care. *PLoS One* 2019;14(6):e0218369. doi: 10.1371/journal.pone.0218369 [published Online First: 2019/06/28]
7. Cohen MS, Shaw GM, McMichael AJ, et al. Acute HIV-1 Infection. *N Engl J Med* 2011;364(20):1943-54. doi: 10.1056/NEJMra1011874 [published Online First: 2011/05/20]
8. Sanders EJ, Agutu C, van der Elst E, et al. Effect of an opt-out point-of-care HIV-1 nucleic acid testing intervention to detect acute and prevalent HIV infection in symptomatic adult outpatients and reduce HIV transmission in Kenya: a randomized controlled trial. *HIV Med* 2022;23(1):16-28. doi: 10.1111/hiv.13157 [published Online First: 20210825]
9. Hamilton DT, Agutu C, Babigumira JB, et al. Modeling the Impact of HIV-1 Nucleic Acid Testing Among Symptomatic Adult Outpatients in Kenya. *J Acquir Immune Defic Syndr* 2022;90(5):553-61. doi: 10.1097/qai.0000000000003013 [published Online First: 20220505]
10. Graham SM, Agutu C, van der Elst E, et al. A Novel HIV-1 RNA Testing Intervention to Detect Acute and Prevalent HIV Infection in Young Adults and Reduce HIV Transmission in Kenya: Protocol for a Randomized Controlled Trial. *JMIR research protocols* 2020;9(8):e16198. doi: 10.2196/16198 [published Online First: 2020/08/09]
11. Sanders EJ, Agutu C, van der Elst E, et al. Effect of an opt-out point-of-care HIV-1 nucleic acid testing intervention to detect acute and prevalent HIV infection in symptomatic adult outpatients and reduce HIV transmission in Kenya: a randomized controlled trial. *HIV Med* 2021 doi: 10.1111/hiv.13157 [published Online First: 2021/08/26]
12. Sanders EJ, Wahome E, Powers KA, et al. Targeted screening of at-risk adults for acute HIV-1 infection in sub-Saharan Africa. *AIDS* 2015;29 Suppl 3(0 3):S221-S30. doi: 10.1097/QAD.0000000000000924
13. Kenya Ministry of Health. National AIDS and STI Control Programme. Kenya HIV Estimates, Report 2018. Accessed on 06 29 2020 at https://nacc.or.ke/wp-content/uploads/2018/11/HIV-estimates-report-Kenya-20182.pdf.
14. Agutu CA, Oduor TH, Kombo BK, et al. High patient acceptability but low coverage of provider-initiated HIV testing among adult outpatients with symptoms of acute infectious illness in coastal Kenya. *PloS one* 2021;16(2):e0246444-e44. doi: 10.1371/journal.pone.0246444
15. The Kenya HIV Testing Services Guidelines. Accessed on June 14th 2020 at https://www.fast-trackcities.org/sites/default/files/Kenya%20HIV%20Testing%20Services%20Guidelines%20%282015%29.pdf.
16. Primary health care systems (PRIMASYS): case study from Kenya, abridged version. Geneva: World Health Organization; 2017. Licence: CC BY-NC-SA 3.0 IGO.

For peer review only - http://bmjopen.bmj.com/site/about/guidelines.xhtml

17. Sharma M, Smith JA, Farquhar C, et al. Assisted partner notification services are cost-effective for decreasing HIV burden in western Kenya. *AIDS* 2018;32(2):233-41. doi: 10.1097/QAD.0000000000001697
18. Irungu EM, Sharma M, Maronga C, et al. The Incremental Cost of Delivering PrEP as a Bridge to ART for HIV Serodiscordant Couples in Public HIV Care Clinics in Kenya. *AIDS Res Treat* 2019;2019:4170615-15. doi: 10.1155/2019/4170615
19. Kenya National Bureau of Statistics. Kenya Data Portal. Consumer Price Indices (CPI) and Inflation Rates for Kenya. Accessed on 11/03/2020 at https://kenya.opendataforafrica.org/ltziiob/consumer-price-indices-cpi-and-inflation-rates-of-kenya.
20. Neumann PJ, Sanders GD, Russell LB, Siegel JE, and Ganiats TG (Eds). Cost-Effectiveness in Health and Medicine (Second Edition. Oxford University Press, 2017. .
21. Salomon JA, Haagsma JA, Davis A, et al. Disability weights for the Global Burden of Disease 2013 study. *The Lancet Global Health* 2015;3(11):e712-e23. doi: 10.1016/S2214-109X(15)00069-8
22. World Bank. The World Fact Book. Kenya Per-Capita Gross Domestic in Current Dollars. Accessed at https://data.worldbank.org/indicator/NY.GDP.PCAP.CD on 4th June 2021.
23. Schwartz NRM, Flanagan MR, Babigumira JB, et al. Cost-Effectiveness Analysis of Adjuvant Neratinib Following Trastuzumab in Early-Stage HER2-Positive Breast Cancer. *J Manag Care Spec Pharm* 2019;25(10):1133-39. doi: 10.18553/jmcp.2019.25.10.1133
24. Babigumira JB, Castelnuovo B, Stergachis A, et al. Cost effectiveness of a pharmacy-only refill program in a large urban HIV/AIDS clinic in Uganda. *PLoS One* 2011;6(3):e18193. doi: 10.1371/journal.pone.0018193 [published Online First: 20110328]
25. Husereau D, Drummond M, Petrou S, et al. Consolidated Health Economic Evaluation Reporting Standards (CHEERS) statement. *BMJ : British Medical Journal* 2013;346:f1049. doi: 10.1136/bmj.f1049
26. Shillcutt SD, Walker DG, Goodman CA, et al. Cost effectiveness in low- and middle-income countries: a review of the debates surrounding decision rules. *Pharmacoeconomics* 2009;27(11):903-17. doi: 10.2165/10899580-000000000-00000
27. Newall AT, Jit M, Hutubessy R. Are Current Cost-Effectiveness Thresholds for Low- and Middle-Income Countries Useful? Examples from the World of Vaccines. *Pharmacoeconomics* 2014;32(6):525-31. doi: 10.1007/s40273-014-0162-x
28. Daroudi R, Akbari Sari A, Nahvijou A, et al. Cost per DALY averted in low, middle- and high-income countries: evidence from the global burden of disease study to estimate the cost-effectiveness thresholds. *Cost Effectiveness and Resource Allocation* 2021;19(1):7. doi: 10.1186/s12962-021-00260-0
29. Ochalek J, Lomas J, Claxton K. Estimating health opportunity costs in low-income and middle-income countries: a novel approach and evidence from cross-country data. *BMJ Global Health* 2018;3(6):e000964. doi: 10.1136/bmjgh-2018-000964
30. Woods B, Revill P, Sculpher M, et al. Country-Level Cost-Effectiveness Thresholds: Initial Estimates and the Need for Further Research. *Value in Health* 2016;19(8):929-35. doi: 10.1016/j.jval.2016.02.017
31. Han X, Xu J, Chu Z, et al. Screening acute HIV infections among Chinese men who have sex with men from voluntary counseling & testing centers. *PLoS One* 2011;6(12):e28792. doi: 10.1371/journal.pone.0028792 [published Online First: 2011/12/24]
32. Long EF. HIV screening via fourth-generation immunoassay or nucleic acid amplification test in the United States: a cost-effectiveness analysis. *PLoS One* 2011;6(11):e27625. doi: 10.1371/journal.pone.0027625 [published Online First: 2011/11/24]

For peer review only

For peer review only - http://bmjopen.bmj.com/site/about/guidelines.xhtml

33. Hoenigl M, Graff-Zivin J, Little SJ. Costs per Diagnosis of Acute HIV Infection in Community-based Screening Strategies: A Comparative Analysis of Four Screening Algorithms. *Clin Infect Dis* 2016;62(4):501-11. doi: 10.1093/cid/civ912 [published Online First: 2015/10/29]
34. Cipriano LE, Zaric GS, Holodniy M, et al. Cost effectiveness of screening strategies for early identification of HIV and HCV infection in injection drug users. *PLoS One* 2012;7(9):e45176. doi: 10.1371/journal.pone.0045176 [published Online First: 2012/10/03]
35. Alere™ Determine™ HIV–1/2 Ag/Ab Combo https://www.fda.gov/media/86959/download.
36. FIRST RESPONSE ®HIV 1-2.O CARD TEST. Rapid Immunochromatographic Card Test for the detection of Antibodies to HIV 1 & 2 in Human Whole Blood/Serum/Plasma. https://www.who.int/diagnostics_laboratory/evaluations/pq-list/hiv-rdts/161220_amended_final_pqpr_0018_010_00_v3.pdf?ua=1.
37. Bwana P, Ageng'o J, Mwau M. Performance and usability of Cepheid GeneXpert HIV-1 qualitative and quantitative assay in Kenya. *PloS one* 2019;14(3):e0213865-e65. doi: 10.1371/journal.pone.0213865
38. Cherutich P, Golden MR, Wamuti B, et al. Assisted partner services for HIV in Kenya: a cluster randomised controlled trial. *The lancet HIV* 2017;4(2):e74-e82. doi: 10.1016/s2352-3018(16)30214-4 [published Online First: 2016/12/04]
39. Government of Kenya. Energy and Petroleum Regulatory Authority. Approved Electricity Charge Rates 2018/2019. (Small COmmercial Customer). Accessed on 08/19/2020 at https://www.epra.go.ke/wp-content/uploads/2018/06/Approved-Tariffs-Aug-2018.pdf.
40. The Global Fund. HIV Viral Load and Early Infant Diagnosis Selection and Procurement Information Tool. Version 2 April 2017. Available at https://www.theglobalfund.org/media/5765/psm_viralloadearlyinfantdiagnosis_content_en.pdf .
41. Walusimbi S, Kwesiga B, Rodrigues R, et al. Cost-effectiveness analysis of microscopic observation drug susceptibility test versus Xpert MTB/Rif test for diagnosis of pulmonary tuberculosis in HIV patients in Uganda. *BMC Health Serv Res* 2016;16(1):563. doi: 10.1186/s12913-016-1804-9 [published Online First: 2016/10/12]
42. National AIDS & STI Control Program, Ministry of Health Kenya. National Quantification report for HIV Commodities for FY 2019/20 to 2021/22, October, 2019.

For peer review only

Table 1. Model inputs: probabilities, outcomes estimates, resource estimates, and cost parameters

	Base case	Low	High	Reference
**Probabilities**				
Provider-recommended HIV Ab testing	0.256	0.205	0.307	Primary data[9]
Opt-out				
Of physician-recommended Ab test (PITC)	0.000	—	—	Primary data[9]
Of scaled-up Ab test (SU-PITC)	0.051	0.041	0.061	Primary data[9]*
Of RNA test (POC-NAAT)	0.051	0.041	0.061	Primary data[9]
HIV prevalence in Kenya	0.049	0.041	0.210	[13]
Linkage to care				
PITC	0.692	0.554	0.830	Primary data[9]
POC-NAAT/SU-PITC	0.892	0.714	1.000	Primary data[9]*
Partner disclosure				
PITC	0.75	0.600	0.900	Primary data[9]
POC-NAAT /SU-PITC	0.67	0.536	0.804	Primary data[9]*
Partner positivity				
If true positive	0.230	0.184	0.276	[38]
If false positive	0.049	0.041	0.210	[13]¶
**Disability weights**				
HIV (symptomatic, pre-AIDS)	0.274	0.184	0.377	[21]
HIV/AIDS (receiving ART)	0.078	0.052	0.111	[21]
**Cost inputs**				
GeneXpert				
Electricity consumption (VA)	824	—	—	Cepheid
Cost per unit (KWA) of electricity	$0.14	$0.07	$0.29	[39]
Kenya voltage	240	—	—	Standard
Power factor	0.6	0.48	0.72	Assumption
Machine (GeneXpert IV)	$17,000	$13,600	$20,400	[40]
Warranty	$6,480	$5,184	$7,776	[40]
Cartridge (per test)	$16.80	$13.44	$20.16	[40]
Maintenance (per year)	$1,800	$1,440	$2,160	[40]
Useful life (years)	5	4	6	[41]
Scrap value	$0	—	—	Assumption
Tests per hour	2.5	2.0	3.0	[40]
Annual run time (hours)	2848	2278	3418	Assumption
Determine	$0.82	$0.41	$1.64	[42]
First Response	$0.73	$0.36	$1.46	[42]
Personnel (cadres) by activity				
Clinical consultation				
Medical officers	12.5%	8%	12%	Primary data
Clinical officers	87.5%	72%	100%	Primary data
Lab testing				
Lab technologist	95%	76%	100%	Primary data
Medical lab officer	5%	4%	6%	Primary data
Pharmacy services				

For peer review only - http://bmjopen.bmj.com/site/about/guidelines.xhtml

Clinical officer	5%	4%	6%	Primary data
Pharmacist	10%	8%	12%	Primary data
Pharmacy technologist	85%	68%	100%	Primary data
Time use (minutes)				
Observation arm				
Initial consultation	4	3	5	Primary data
Lab testing	54	31	85	Primary data
Post-lab consultation	4	1	8	Primary data
Pharmacy	2	1	5	Primary data
Intervention arm				
Initial consultation	3	3	5	Primary data
Pre-test counseling	17	14	22	Primary data
Lab testing	68	45	93	Primary data
GeneXpert testing	110	101	144	Primary data
Post-test counseling	3	2	4	Primary data
Post-lab consultation	8	5	14	Primary data
Pharmacy	5	3	7	Primary data
Wages (per hour)				
Medical officers	$1.04	$0.39	$1.56	Primary data[π]
Clinical officers	$0.50	$0.33	$0.75	Primary data[π]
Lab technologist	$0.36	$0.18	$0.54	Primary data[π]
Medical lab officer	$0.47	$0.36	$0.71	Primary data[π]
Pharmacist	$1.08	$0.39	$1.62	Primary data[π]
Pharmacy technologist	$0.35	$0.33	$0.52	Primary data[π]
Counselor	$0.47	$0.33	$0.71	Primary data[π]
Linkage to HIV care[&]	$225.45	$112.73	$338.18	[17]
Antiretroviral therapy				
Initiation	$102.54	$51.27	$153.81	[17]
Maintenance (annual)	$351.10	$175.55	$526.65	[17]
Discounted LYs (10-year time horizon)	8.53			Calculated
Pre-exposure prophylaxis (PrEP) (annual)	$111.29	$83.47	$166.94	[18]
Duration of PrEP (years)	0.315	0.252	0.378	Kenya MOH
Acute partner services (index positive)	$114.39	$57.20	$171.59	[17]

* Assumption: opt out rate, rate pf linkage to care, and rate of partner disclosure in the SU-PITC arm are equivalent to rates in the POC-NAAT arm

¶ Assumption: equal to HIV prevalence

π Lower estimate equals starting wage for cadre

& Cost of pre-ART laboratory tests assuming all patients have a CD4 cell count of over 350 cells/μl

For peer review only

For peer review only - http://bmjopen.bmj.com/site/about/guidelines.xhtml

Table 2. Mean base case patient costs and outcomes of HIV testing strategies to detect acute and prevalent HIV infection in adult outpatients seeking healthcare for symptoms compatible with acute HIV infection in Kenya

	PITC	SU-PITC	POC-NAAT
Costs			
Testing costs	$0.50	$1.87	$29.83
HIV-positive patient management	$46.74	$171.72	$185.08
Total costs	$47.25	$173.59	$214.91
Incremental cost	—	$126.34	$41.32
Testing			
Proportion accurately classified[≠]	0.2530	0.9374	0.9488
Incremental accurate classification	—	0.6844	0.0114
Cost per additional accurately classified case	—	$184.59	$3,619
HIV positive case yield	0.0125	0.0460	0.0465
Incremental HIV-positive case yield	—	0.0335	0.0005
Cost per additional HIV-positive case identified	—	$3,774	$83,053
HIV prevention			
HIV cases (per 10,000)	606	596	545
HIV cases averted	—	10	51
Cost per HIV case averted	—	Dominated*	$2.75[¶]
HIV cases averted (reference to PITC)	—	10	61
Cost per HIV case averted (reference to PITC)	—	$12.63	$2.75
Main outcome			
DALYs	0.2140	0.1757	0.1598
DALYs averted	—	0.0382	0.0159
Cost per DALY averted	—	Dominated*	$3,098[¶]
DALYs averted (reference to PITC)	—	0.0382	0.0541
Cost per DALY averted (reference to PITC)	—	$3,306	$3,098

*SU-PITC is excluded from the incremental analysis by extended dominance

¶ICER compares POC-NAAT to PITC

≠A factor of both the decision to test and diagnostic test accuracy

For peer review only

For peer review only - http://bmjopen.bmj.com/site/about/guidelines.xhtml

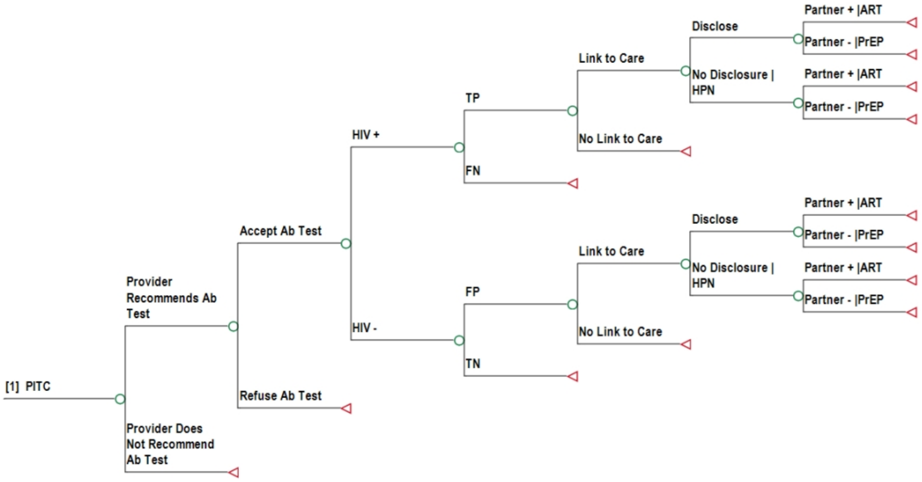

108x60mm (300 x 300 DPI)

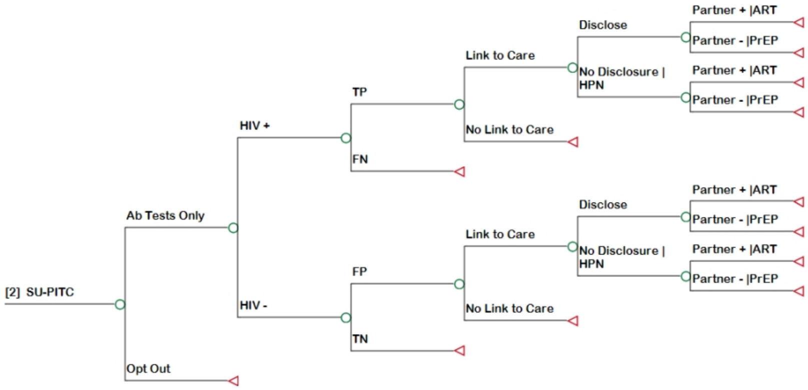

108x60mm (300 x 300 DPI)

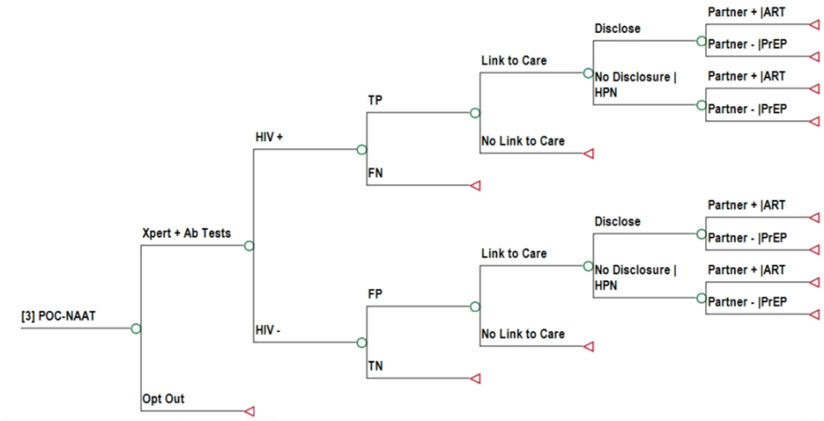

108x60mm (300 x 300 DPI)

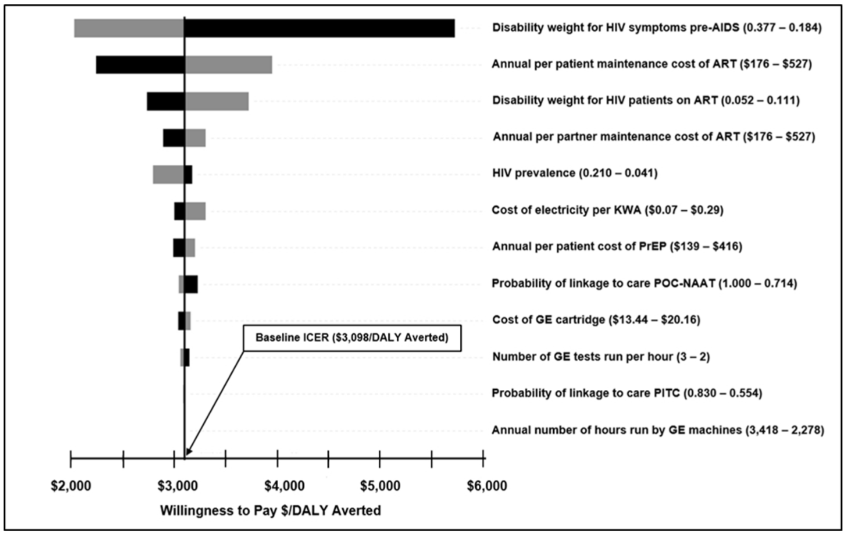

108x60mm (300 x 300 DPI)

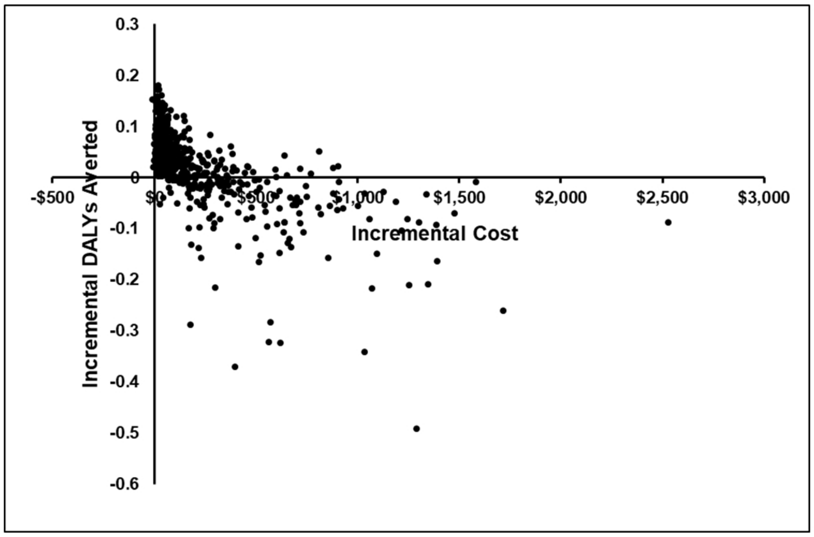

108x60mm (300 x 300 DPI)

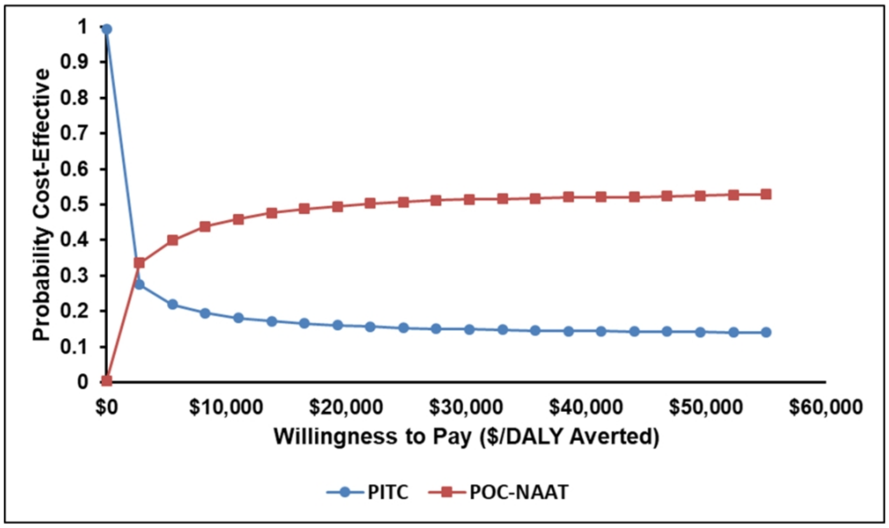

108x60mm (300 x 300 DPI)

Time and Motion Survey

Date: |__ __|__ __|__ __ __ __|

Clinic: |____

Patient #: |__ __ __|

Note: Please use the 24 hour clock system (use time piece with 24-hour clock for this study)

#	Point of patient contact	Time in	Time out	Signature
1	**Patient enters clinic**<br>Received by (initial): |__________|	|___ ___:___ ___|		
2	**Patient seen by (designation): |________________|**<br>Initial: |__________|	|___ ___:___ ___|	|___ ___:___ ___|	
3	**Patient seen by (designation): |________________|**<br>Initial: |__________|	|___ ___:___ ___|	|___ ___:___ ___|	
4	**Patient seen by (designation): |________________|**<br>Initial: |__________|	|___ ___:___ ___|	|___ ___:___ ___|	
5	**Patient seen by (designation): |________________|**<br>Initial: |__________|	|___ ___:___ ___|	|___ ___:___ ___|	
6	**Patient seen by (designation): |________________|**<br>Initial: |__________|	|___ ___:___ ___|	|___ ___:___ ___|	
7	**Patient seen by (designation): |________________|**<br>Initial: |__________|	|___ ___:___ ___|	|___ ___:___ ___|	
8	**Patient seen by (designation): |________________|**<br>Initial: |__________|	|___ ___:___ ___|	|___ ___:___ ___|	
9	**Patient seen by (designation): |________________|**<br>Initial: |__________|	|___ ___:___ ___|	|___ ___:___ ___|	
15	**Patient leaves clinic**<br>Seen off by (initial): |__________|		|___ ___:___ ___|	

For peer review only

CHEERS Checklist

Testing strategies to detect acute and prevalent HIV infection in adult outpatients seeking healthcare for symptoms compatible with acute HIV infection in Kenya: a cost-effectiveness analysis

	Item #	Recommendation	Reported on page #/Line # Comments
**Title and abstract**			
Title	1	Identify the study as an economic evaluation, or use more specific terms such as "cost-effectiveness analysis" and describe the interventions compared.	Page 1, Lines 1,2,3
Abstract	2	Provide a structured summary of objectives, perspective, setting, methods (including study design and inputs), results (including base case and uncertainty analyses), and conclusions.	Page 2, Lines 33 – 57
**Introduction**			
Background and objectives	3	Provide an explicit statement of the broader context for the study.	Page 4, Lines 94 - 107
		Present the study question and its relevance for health policy or practice decisions.	Page 5, Lines 121 - 124
**Methods**			
Target population and subgroups	4	Describe characteristics of the base case population and subgroups analyzed, including why they were chosen.	Page 5, Lines 134 - 139
Setting and location	5	State relevant aspects of the system(s) in which the decision(s) need(s) to be made.	Page 5, Lines 141 - 145
Study perspective	6	Describe the perspective of the study and relate this to the costs being evaluated.	Page 7, Lines 215 - 220
Comparators	7	Describe the interventions or strategies being compared and state why they were chosen.	Pages 5, Lines 147 - 163
Time horizon	8	State the time horizon(s) over which costs and consequences are being evaluated and say why appropriate.	Page 5, Lines 165 - 169
Discount rate	9	Report the choice of discount rate(s) used for costs and outcomes and say why appropriate.	Page 7 Line 251
Choice of health outcomes	10	Describe what outcomes were used as the measure(s) of benefit in the evaluation and their relevance for the type of analysis performed.	Page 6, Lines 202 - 213
Measurement of effectiveness	11a	Single study-based estimates: Describe fully the design features of the single effectiveness study and why the single study was a	The analysis was a model-based analysis with data from multiple sources and did not

For peer review only

		sufficient source of clinical effectiveness data.	rely on a single effectiveness study.
	11b	Synthesis-based estimates: Describe fully the methods used for identification of included studies and synthesis of clinical effectiveness data.	Because of the paucity of data for model parametrization, we did not perform a formal synthesis of data on any single parameter (i.e., systematic review or metanalysis). We used the best available evidence as judged by our team. We combined this with data from primary analyses to fully parametrize the decision model.
Measurement and valuation of preference-based outcomes	12	If applicable, describe the population and methods used to elicit preferences for outcomes.	We used disability weights or adjust for quality of life and not utilities. As such, we did not elicit preferences for outcomes or use existing preference-based studies for utility estimation.
Estimating resources and costs	13a	Single study-based economic evaluation: Describe approaches used to estimate resource use associated with the alternative interventions. Describe primary or secondary research methods for valuing each resource item in terms of its unit cost. Describe any adjustments made to approximate to opportunity costs.	The study was a model-based study using estimates from both primary analysis of time use and wage data and published estimates for the cost inputs. Therefore, we did not rely on a single study for estimation of resource use and cost.
	13b	Model-based economic evaluation: Describe approaches and data sources used to estimate resource use associated with model health states. Describe primary or secondary research methods for valuing each resource item in terms of its unit cost. Describe any adjustments made to approximate to opportunity costs.	See table 1, Pages 17 and 18, Lines 606 - 613
Currency, price date, and conversion	14	Report the dates of the estimated resource quantities and unit costs. Describe methods for adjusting estimated unit costs to the year of reported costs if necessary. Describe methods for converting costs into a common currency base and the exchange rate.	Page 7, Lines 246- 249
Choice of model	15	Describe and give reasons for the specific type of decision-analytical model used. Providing a figure to show model structure is strongly recommended	Page 6, Pages 171 - 213; Tree diagram is Figure 1

For peer review only

Assumptions	16	Describe all structural or other assumptions underpinning the decision-analytical model.	Page 6, Lines 190 and 194
Analytic methods	17	Describe all analytical methods supporting the evaluation. This could include methods for dealing with skewed, missing, or censored data; extrapolation methods; methods for pooling data; approaches to validate or make adjustments (such as half cycle corrections) to a model; and methods for handling population heterogeneity and uncertainty.	We include details of the approach to statistical analysis under the Statistical Analysis section on page 7 & 8 (lines 256 – 291), including the handling of uncertainty. Our study did not include pre-specified sub-group analyses and therefore did not include an analysis of heterogeneity.
**Results**			
Study parameters	18	Report the values, ranges, references, and if used, probability distributions for all parameters. Report reasons or sources for distributions used to represent uncertainty where appropriate. Providing a table to show the input values is strongly recommended.	See table 1, Pages 17 and 18, Lines 606 - 613
Incremental costs and outcomes	19	For each intervention, report mean values for the main categories of estimated costs and outcomes of interest, as well as mean differences between the comparator groups. If applicable, report incremental cost-effectiveness ratios.	Table 2, Page 19, Lines 615-620
Characterizing uncertainty	20a	Single study–based economic evaluation: Describe the effects of sampling uncertainty for estimated incremental cost, incremental effectiveness, and incremental cost-effectiveness, together with the impact of methodological assumptions (such as discount rate, study perspective).	The study was a model-based study using estimates from multiple sources. Therefore, we did not consider the effects of sampling uncertainty on incremental estimates.
	20b	Model-based economic evaluation: Describe the effects on the results of uncertainty for all input parameters, and uncertainty related to the structure of the model and assumptions.	See results of univariate and probabilistic sensitivity analysis on page 9 and 10 (lines 340-360).
Characterizing heterogeneity	21	If applicable, report differences in costs, outcomes, or cost-effectiveness that can be explained by variations between subgroups of patients with different baseline characteristics or other observed variability in effects that are not reducible by more information.	Our study did not include pre-specified sub-group analyses and therefore we did not need to assess heterogeneity.
**Discussion**			

For peer review only

Study findings, limitations, generalizability, and current knowledge	22	Summarize key study findings and describe how they support the conclusions reached. Discuss limitations and the generalizability of the findings and how the findings fit with current knowledge.	Page 10, Lines 354 - 364
**Other**			
Source of funding	23	Describe how the study was funded and the role of the funder in the identification, design, conduct, and reporting of the analysis. Describe other nonmonetary sources of support.	Page 12 and 13, Lines 472 - 482
Conflicts of interest	24	Describe any potential for conflict of interest among study contributors in accordance with journal policy. In the absence of a journal policy, we recommend authors comply with International Committee of Medical Journal Editors' recommendations.	Page 13, Lines 485 - 486

For peer review only
